# Positive Selection of Deleterious Alleles through Interaction with a Sex-Ratio Suppressor Gene in African Buffalo: A Plausible New Mechanism for a High Frequency Anomaly

**DOI:** 10.1371/journal.pone.0111778

**Published:** 2014-11-05

**Authors:** Pim van Hooft, Ben J. Greyling, Wayne M. Getz, Paul D. van Helden, Bas J. Zwaan, Armanda D. S. Bastos

**Affiliations:** 1 Resource Ecology Group, Wageningen University, Wageningen, The Netherlands; 2 Mammal Research Institute, Department of Zoology & Entomology, University of Pretoria, Hatfield, South Africa; 3 Agricultural Research Council, Irene, South Africa; 4 Department of Environmental Science Policy & Management, University of California, Berkeley, California, United States of America; 5 School of Mathematical Sciences, University of KwaZulu-Natal, Durban, South Africa; 6 DST/NRF Centre of Excellence for Biomedical TB Research, US/MRC Centre for TB Research, Division of Molecular Biology and Human Genetics, Faculty of Health Sciences, Stellenbosch University, Tygerberg, South Africa; 7 Laboratory of Genetics, Wageningen University, Droevendaalsesteeg 1, Wageningen, The Netherlands; Swiss Federal Institute of Technology (ETH Zurich), Switzerland

## Abstract

Although generally rare, deleterious alleles can become common through genetic drift, hitchhiking or reductions in selective constraints. Here we present a possible new mechanism that explains the attainment of high frequencies of deleterious alleles in the African buffalo (*Syncerus caffer*) population of Kruger National Park, through positive selection of these alleles that is ultimately driven by a sex-ratio suppressor. We have previously shown that one in four Kruger buffalo has a Y-chromosome profile that, despite being associated with low body condition, appears to impart a relative reproductive advantage, and which is stably maintained through a sex-ratio suppressor. Apparently, this sex-ratio suppressor prevents fertility reduction that generally accompanies sex-ratio distortion. We hypothesize that this body-condition-associated reproductive advantage increases the fitness of alleles that negatively affect male body condition, causing genome-wide positive selection of these alleles. To investigate this we genotyped 459 buffalo using 17 autosomal microsatellites. By correlating heterozygosity with body condition (heterozygosity-fitness correlations), we found that most microsatellites were associated with one of two gene types: one with elevated frequencies of deleterious alleles that have a negative effect on body condition, irrespective of sex; the other with elevated frequencies of sexually antagonistic alleles that are negative for male body condition but positive for female body condition. Positive selection and a direct association with a Y-chromosomal sex-ratio suppressor are indicated, respectively, by allele clines and by relatively high numbers of homozygous deleterious alleles among sex-ratio suppressor carriers. This study, which employs novel statistical techniques to analyse heterozygosity-fitness correlations, is the first to demonstrate the abundance of sexually-antagonistic genes in a natural mammal population. It also has important implications for our understanding not only of the evolutionary and ecological dynamics of sex-ratio distorters and suppressors, but also of the functioning of deleterious and sexually-antagonistic alleles, and their impact on population viability.

## Introduction

Deleterious alleles play a central role in inbreeding depression and generally have a negative impact on the genetic viability of populations. Negative (purifying) selection is expected to eliminate deleterious alleles from a population [1]. Deleterious alleles can, however, be maintained in a population through balancing selection or recurrent mutations [2]. Although generally rare, deleterious alleles can become common through reductions in selective constraints on non-synonymous mutations, random genetic drift (especially in small populations) or hitchhiking (linkage to a gene undergoing positive selection, i.e. indirect positive selection) [3], [4].

The role of deleterious alleles in natural populations can be studied by looking at correlations between observed heterozygosity of autosomal microsatellites and various traits that are expected to affect fitness, such as body condition, disease status and survival. These heterozygosity-fitness correlations (HFCs) have been documented in a wide range of vertebrates [5], [6], although most of these studies do not measure fitness directly. Low fitness is largely attributed to increased homozygosity of recessive deleterious alleles [5], [7].

Two non-mutually exclusive hypotheses have been put forward to explain HFCs [2], [7]. The *general effects hypothesis* postulates that HFCs are due to inbreeding depression, which results from negative correlations between genome-wide heterozygosity and the inbreeding coefficient. Specifically, inbreeding affects all loci in an individual, resulting in a lower heterozygous fraction of both microsatellites and expressed genes among inbred individuals. The *local effects hypothesis* postulates that HFCs are a consequence of physical linkage between specific microsatellites and expressed genes, resulting in linkage disequilibrium (LD). Although the general effects hypothesis is currently considered to explain most occurrences of HFCs in natural populations, many of these analyses are unable to convincingly discriminate between the two hypotheses [5], [7].

HFCs related to bovine tuberculosis (BTB) status have been observed in wild boar (*Sus scrofa*) and were attributed to a combination of local LD and general inbreeding effects [8]. BTB is also present in the African buffalo (*Syncerus caffer*) population of the Kruger National Park in South Africa, where prevalence is increasing over time. In 1991–1992 BTB was observed only in southern Kruger (south of the Olifants River), with a herd prevalence of 4–27% (highest frequency in the southern-most section, which is south of the Sabie River). In 1998, the disease was still mainly restricted to southern Kruger, but herd prevalence had increased to 16–38%. In contrast, the overall prevalence of 1.5% in northern Kruger was based on infected individuals from a single herd just north of the Olifants River [9]. In 2005, the prevalence increased to 28–45% in southern Kruger and the disease reached the northern-most border of the park [10], [11].

In this study, we used autosomal microsatellite data (17 microsatellites) from Kruger buffalo sampled in 1998 (459 DNA samples) to determine whether BTB-related HFCs exist in this population, by contrasting the essentially BTB-negative northern region with the BTB-positive southern region. We hypothesized low heterozygosity among BTB-positive individuals and among individuals of low body condition (LBC) relative to those of high body condition (HBC). We included body condition because BTB infection is generally associated with LBC, as has also been observed in the Kruger buffalo population [12]. Our aim was to obtain greater insights into the characteristics and dynamics of deleterious alleles underlying HFCs. This we did by analyzing how multilocus heterozygosities, their constituent single-locus heterozygosities and microsatellite allele frequency dynamics were related to disease status, body-condition status, sex and locality (allele clines, essentially BTB-free northern *vs.* BTB-infected southern Kruger). We also tested whether there was an interaction between HFCs and Y-chromosomal sex-ratio distorters (genes causing unequal primary sex ratio through differences in number or quality of X- and Y-bearing spermatozoa) and suppressors (genes suppressing sex-ratio distorter activity), whose occurrence in the Kruger buffalo population has been suggested in a previous study [13]. Sex-ratio distorters, in particular, can have a strong negative effect on fitness by decreasing fertility [14], [15]. Sex-ratio distorters and suppressors may thereby affect HFCs, particularly because these genes have previously been associated with body condition in the Kruger buffalo population [13].

In this study, we observed HFCs and associations between particular microsatellite alleles and body condition, as well as a direct association between autosomal genetic parameters and a Y-chromosomal haplotype, using novel statistical methods. We additionally hypothesized an underlying mechanism to explain our findings.

## Materials and Methods

### Ethics

The blood samples used in this study are by-products from BTB prevalence studies [9], [16] in which 20–30 animals were sampled per herd. Ten herds from each of three geographic zones (north of Olifants River; between Olifants and Sabie River; south of Sabie River) were targeted to maximize the precision of the prevalence estimate at the herd and population level, while keeping mortalities to a minimum [9]. Animals were culled using techniques approved by Kruger National Park authorities [17].

### Description of population and samples

Kruger National Park in South Africa is a 19,485 km^2^ wildlife reserve. Its African buffalo population varies around 30,000 individuals. The average breeding herd size is about 250 individuals (range 30–1100). Blood samples were collected from 660 culled individuals from 32 herds in September-November 1998, which is at the junction of the dry (April-October) and wet (November-March) seasons. Herds were sampled at random from breeding herds counted in the annual Kruger census with individuals selected without regard to age or sex [9]. BTB tests were conducted in 243 individuals from 12 herds, north of the Olifants River, and in 411 individuals, from 20 herds, from the southern region of Kruger. Age was estimated in years. Individuals were grouped into LBC and HBC classes using a standardized index score that ranged from 0 to 3 for the LBC class and from 4 to 5 for the HBC class, based on fat deposits along the back and rump (number of genotyped individuals per index score; 0: 1, 1: 2, 2: 9, 3: 286, 4: 160, 5: 0) [18]. These classes represent body condition at the end of the dry and beginning of the wet season. Detailed protocols of BTB diagnosis, age estimation and body condition scoring have been described elsewhere [9], [13], [18], [19].

### Genetic analyses

DNA was isolated from 459 individuals, 138 from northern and 321 from southern Kruger, randomly selected from 30 of the 32 herds (no blood samples were available from two herds from northern Kruger; map with sampling localities: Figure S1). The following 17 autosomal microsatellites were analysed [20]: *BM1824* (number of alleles: 14), *BM3205* (14), *BM3517* (7), *BM4028* (4), *BM719* (14), *CSSM19* (12), *DIK020* (19), *ETH10* (3), *ETH225* (4), *ILSTS026* (11), *INRA006* (9), *INRA128* (7), *SPS115* (16), *TGLA159* (9), *TGLA227* (4), *TGLA263* (6) and *TGLA57* (10). These microsatellites were randomly chosen with respect to genomic location in cattle [20]. Only four percent of the PCR amplifications (295 out of 7083 (17×459)) were not successful. A detailed laboratory analysis protocol for autosomal microsatellites has been described elsewhere [20].

Y-chromosomal microsatellite data generated in an earlier study [19] were available for 45 males from northern Kruger and 124 from southern Kruger included in this study. A 3-number code was assigned to each Y-chromosomal haplotype, with each number corresponding to a microsatellite allele. Six males from northern Kruger and 40 from southern Kruger carried haplotype 557, which is thought to be linked to a sex-ratio suppressor [13]. A detailed laboratory analysis protocol for the Y chromosome has been described elsewhere [19].

### Statistical analyses

Multilocus estimates of observed heterozygosity (ML-*H*_o_, equation 2.13 in [21]), expected heterozygosity (ML-*H*_e_, equation 2.12 in [21]) and allele frequency were first calculated per locus and subsequently averaged across loci. Standard errors of these multilocus indices were estimated by bootstrapping individuals (1000 samplings), using Excel 2010 [22], and rescaled by multiplying by the ratio of original to bootstrap mean (only a small adjustment as all bootstrap means were close to the original mean). Ninety-five percent confidence intervals of single-locus estimates of *H*_e_ (PL-*H*_e_) were estimated with Arlequin 2.000 [23], [24].

We performed population differentiation tests by permuting genotypes among groups, and tests for Hardy-Weinberg equilibrium by permuting alleles among individuals within groups (herds, subpopulations or other groups of individuals), using FSTAT 2.93 [25], [26]. Analyses of Wright's *F*-statistics according to Weir and Cockerham's method [27], using FSTAT 2.93, and pairwise relatedness (Lynch and Ritland's *r* [28]) per sex or age group, using Spagedi 1.4 [29], were conducted with their standard errors being estimated by jackknifing across loci. Average opposite-sex relatedness per herd and its standard error were calculated with Genaiex 6.5 [30].

Missing genotypes are expected to bias per-individual estimates of ML-*H*_o_ and number of majority alleles (majority alleles: see Results section). To minimize this bias, missing single-locus data were replaced by single-locus estimates of mean observed heterozygosity (PL-*H*_o_) and mean number of majority alleles for northern or southern Kruger. Replacing missing data with average values does not bias mean values per individual and results in conservative *P*-value estimates (Online Table S1 in [7]).

Different alleles at a single locus and different loci in LD cannot be considered as independent data points in statistical analyses, which, if uncorrected for, results in pseudo-replication. To avoid this type of pseudo-replication, most probabilities were obtained by randomizing complete individual multilocus genotypes among groups of individuals (denoted as *P*_randomization_), using Excel 2010. Randomization at the level of individuals cancelled out pseudo-replication at the lower levels (i.e. alleles and loci), because each randomized data set was influenced by the pseudo-replication to the same extent as the original data set. Probabilities were estimated as twice (in order to arrive at a two-sided *P*-value) the fraction of random data sets showing the same or larger value (smaller in case of a negative) of a statistical parameter (mean, adjusted *R* ^2^, Spearman correlation coefficient, χ^2^-value) than the original data set, using 100,000 randomizations.

Close relatedness within herds results in pseudo-replication of genotypes, because genotypes of related individuals are not independent of each other. We controlled for this type of pseudo-replication by generating null models, using Excel 2010, whereby observed and expected counts of alleles were first calculated per herd and then summed across herds. The expected counts assumed equal allele frequencies among classes (e.g. LBC and HBC) within herds, while allowing for allele frequency differences among herds, as well as among classes at the population level because herds were weighted differently for each class in relation to the counts per herd. Allele frequencies based on the summed counts, which essentially are frequencies weighted by sample size per herd, were used for calculating differences in statistical parameters between observed and expected counts (similar to a χ^2^ test). Probabilities were estimated by randomizations of complete multilocus genotypes per herd, using Excel 2010 (100,000 samplings). Herds with only one class were excluded from the calculations because these cannot be permutated. Randomization per herd cancelled out pseudo-replication of genotypes within herds, because herd affiliation remained unchanged and therefore each randomized data set was influenced by the pseudo-replication to the same extent as the original data set. Randomization per herd also corrected for population stratification due to herd structure, because it analysed differences between the original data, which reflected differences both within and among herds, and the null models, which reflected only differences among herds. In logistic regression analyses, which were performed with SPSS 19, correction for population stratification was performed by including the latitude of each herd. We chose to include latitude as a continuous variable rather than herd affiliation as a mixed random factor, because allele clines running in a north-south direction were observed for most of the common alleles (see Results section).

HFCs were analysed by logistic regressions involving body condition (LBC and HBC), disease status (BTB-positive and BTB-negative), ML-*H*_o_, age, sex, and latitude per herd. Additionally, HFCs were studied by analysing the variation of pairwise group (LBC vs. HBC) differences in PL-*H*_e_ among loci. The role of majority alleles (see Results section) in HFCs was analysed by logistic regression involving body condition (LBC and HBC), number of homozygous majority alleles per individual, age, sex and latitude per herd. Additionally, χ^2^ tests were performed for associations between body condition and genotype class (majority allele homozygotes, majority allele heterozygotes, homozygotes with no majority allele, heterozygotes with no majority allele). The role of sexual antagonism in HFCs was analysed by sex-specific LBC-HBC group differences in ML-*H*_e_ and correlations between sexes in LBC-HBC allele frequency differences. The latter correlations were analysed after exclusion of rare alleles with a frequency lower than 0.05 to prevent low sample size bias.

We performed a large number of tests in analysing HFCs, majority alleles and sexual antagonism, some of which are only presented as supporting information. To correct for the large number of tests we calculated Holm-Bonferroni corrected *P*-values using Holm's weighted procedure [31]. The details of these tests together with the corrected *P*-values are provided in Table S1. The results presented in this study are significant after Holm-Bonferroni correction (corrected *P*<0.05), except for the logistic regression between body condition status and ML-*H*_o_.

Two types of statistical test were performed to show the occurrence of positive selection: associations between allele frequency and effect size, with a positive correlation being indicative of positive selection, and correlations between allele frequency and latitude (allele clines). A χ^2^ test was used to test for an association between haplotype status (presence – absence of haplotype 557) and number of homozygous majority alleles.

Baseline values of PL-*H*_e_ and allele frequency per group (e.g. northern Kruger and southern Kruger) or pair of groups (e.g. LBC vs. HBC and BTB-positive vs. BTB-negative) were obtained by pooling individuals from the group(s) involved. Error bars in figures that represent 95% confidence intervals of binomial proportions were estimated according to Wilson [32]. Stouffer's *Z*-test was used for combining probabilities from different independent tests [33]. Forward and backward selection in multiple logistic regression analyses always resulted in the same model. Unless otherwise indicated, *P*-values are two-sided and α-levels are 0.05. Means in the text are reported together with their 95% confidence interval.

The raw data set supporting the results of this article is available from the Dryad Digital Repository: http://doi.org/10.5061/dryad.23d13 (http://datadryad.org/).

## Results

### General genetic structure

Significant LD, based on 2720 random associations (number of randomizations fixed by the nominal level for multiple tests according to the help file of FSTAT, here 0.05), occurred between microsatellite pairs *CSSM19*-*BM1824*, *CSSM19*-*BM3205*, *BM1824*-*BM3205* and *INRA006*-*ILSTS026* (*P* = 0.00037 for each pair, Bonferroni-corrected *α*-level  = 0.00037; overall test with Kruger treated as a single population). LD among *CSSM19, BM1824* and *BM3205* was probably due to physical linkage, since they are located on chromosome 1 in cattle, separated by around 6000 kb [20].

Microsatellites *BM1824*, *TGLA227*, *TGLA159*, *BM4028* and *INRA128* showed significant positive deviations from Hardy-Weinberg equilibrium (*P*<0.003, Bonferroni-corrected *α*-level  = 0.003, herds treated as populations; average *F*_IS_ = 0.097, average *F*_IS_ other microsatellites  = −0.0017). Although these deviations may have been caused by null alleles, they are unlikely to have had a significant influence on our results (Text S1).

Wright's *F*-statistics (using Weir and Cockerham's method [27]) showed only minor genetic differentiation among herds (mean and 95% CI: *F*_ST_ = 0.012±0.004) and subpopulations (*F*_ST_ northern vs. southern Kruger  = 0.005±0.002), and no strong deviation from Hardy-Weinberg equilibrium within herds (*F*_IS_ = 0.021±0.024). The average male, female and opposite-sex relatedness (using Lynch and Ritland's *r* [28]) within herds was low (females: 0.027±0.008, *n* = 276; males: 0.015±0.012, *n* = 183; opposite sex: 0.017±0.006). Also the average relatedness among calves within herds was low (0-year olds: 0.004±0.031, *n* = 76; 1-year olds: 0.009±0.035, *n* = 77; 2-year olds: 0.008±0.031, *n* = 80). Low *F*_IS_ and relatedness values indicate only limited inbreeding due to non-random mating.

### HFCs associated with body condition

In southern Kruger, characterized by high BTB prevalence, there was a significant multiple logistic regression between individual body condition (0: LBC, 1: HBC) and multilocus observed heterozygosity (ML-*H*_o_, positive relationship) together with age (negative relationship) and latitude (positive relationship) (χ^2^-value  = 46.81, *P*_model_  = 3.8×10^−10^, *P*_ML-*H*o_  = 0.048, *P*_age_ = 0.000063, *P*_latitude_ = 0.0000048, *n*_LBC_ = 230, *n*_HBC_ = 90, *n*_herds_ = 20). Sex and BTB status were not significant when added to the regression model (*P*_BTB_ = 0.14, *P*_sex_ = 0.59). In other words, body condition increased with increasing ML-*H*_o_, decreasing age and increasing latitude (higher to the north). However, ML-*H*_o_ did not have a significant effect on BTB status in southern Kruger (*P* = 0.52, *n*_BTB+_ = 86, *n*_BTB-_ = 233, *n*_herds_ = 20) or on body condition in northern Kruger (*P*>0.82, *n*_LBC_ = 68, *n*_HBC_ = 70, *n*_herds_ = 10).

We further explored HFCs with per-locus expected heterozygosity (PL-*H*_e_) per group of buffalo. According to Szulkin et al. 2010 [7], comparing among different loci the relative strength of correlations between per-locus observed heterozygosity (PL-*H*_o_) and fitness-affecting traits is the only appropriate test to differentiate between the general and the local effects hypothesis [7]. However, PL-*H*_o_ has relatively low statistical power because it is only binomial. We performed an alternative test to differentiate between the general and the local effects hypothesis, which is based on PL-*H*_e_. PL-*H*_e_ is derived from allele frequencies and therefore has more degrees of freedom than PL-*H*_o_. It was thus expected that tests involving PL-*H*_e_ to have greater statistical power.

Under the local effects hypothesis, allele frequencies at microsatellites physically linked to expressed genes that influence a certain trait (in our case body condition) can differ between groups delineated according to that trait (in our case LBC vs. HBC). In other words, a deleterious allele at an expressed gene that has a negative effect on body condition is expected to be more frequent among LBC individuals than among HBC individuals. The same would be true for a microsatellite allele linked to such a deleterious allele. Subsequently, differences in microsatellite allele frequencies among groups result in differences in PL-*H*_e_. In contrast, under the general effects hypothesis no differences in microsatellite allele frequencies, and hence no differences in PL-*H*_e_, are expected between groups, irrespective of the average inbreeding coefficient of these groups. This is because inbreeding does not affect frequencies of alleles (page 33-34 in [21]) that are not linked to the trait according to which the groups are delineated. The absence of such a linkage is a key assumption in the general effects hypothesis [7].

Also genetic drift due to population stratification can result in differences in PL-*H*_e_. Genetic drift is influenced by the effective size of groups and by the amount of gene flow among groups. It is possible to differentiate between effects of genetic drift on heterozygosity and HFCs by comparing group differences in PL-*H*_e_ among loci. The decrease in PL*-H*_e_ of a group from one generation to the next (PL-*H*_e,t_ minus PL-*H*_e,t-1_) is expected to be proportional to the probability that two randomly sampled alleles coalesce in the previous generation (related to effective group size and expected to be the same for each microsatellite) multiplied by PL-*H*_e_ in the previous generation (equation 3.51 in [21]). The largest increases over time in the difference in PL-*H*_e_ between two groups (PL-*H*_e,group-i_ minus PL-*H*_e,group-j_) are therefore expected for those microsatellites with the highest baseline levels of PL-*H*_e_. The decrease in PL-*H*_e_ at the group level relative to the population as a whole is determined by the number of migrant alleles among groups (expected to be the same for each microsatellite), i.e. by *F*_ST_ multiplied by PL-*H*_e_ at the population level (equation 4.15 in [21]). It follows from this equation that, again, the largest increases over time in the difference in PL-*H*_e_ between two groups (PL-*H*_e,group-i_ minus PL-*H*_e, group-j_) are expected for those microsatellites with the highest baseline levels of PL-*H*_e_.

**Figure 1:**
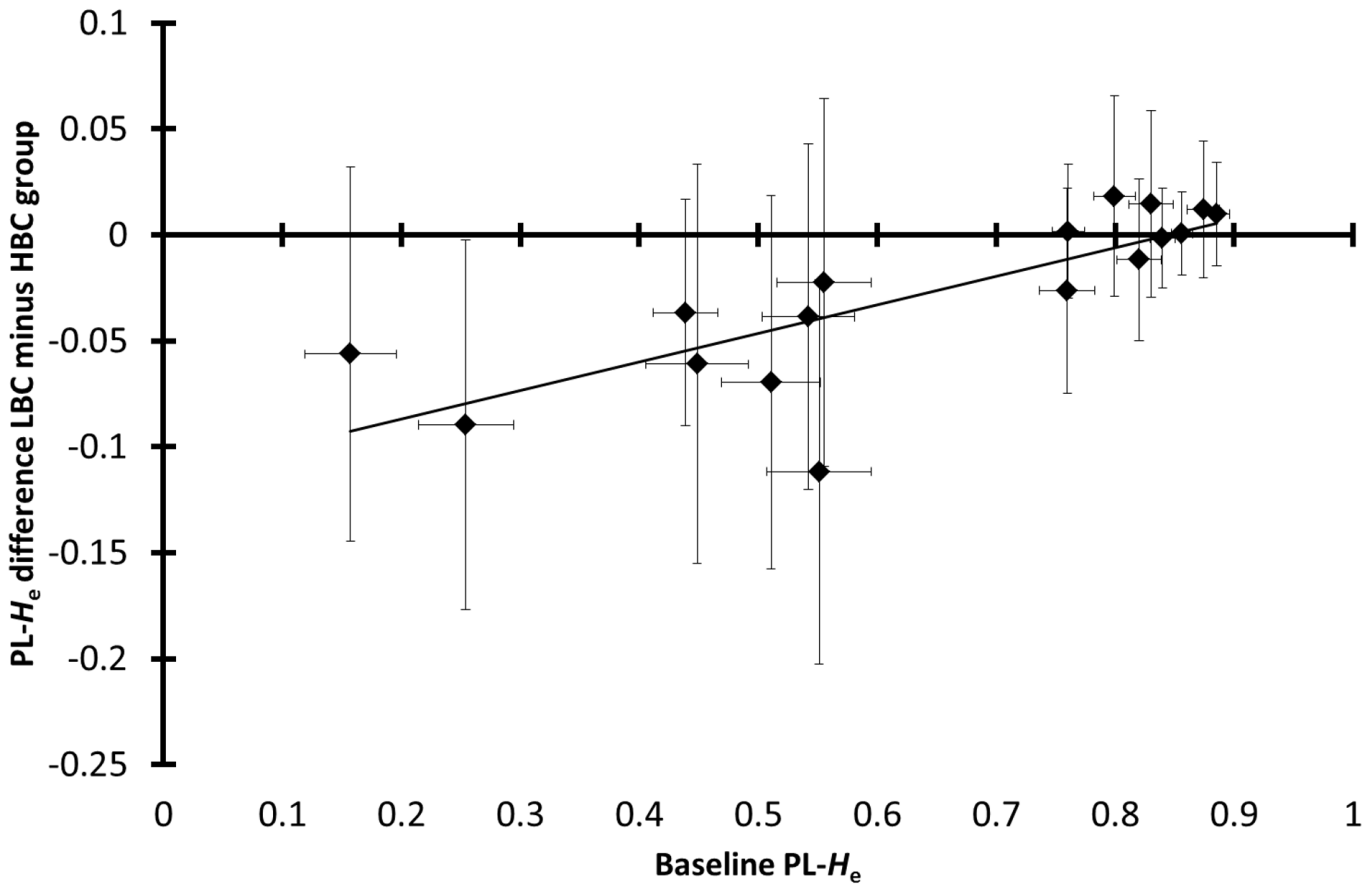
Correlation between LBC-minus-HBC group difference in PL-*H*_e_ and baseline PL-*H*_e_. Baseline PL-*H*_e_ (expected heterozygosity per locus): PL-*H*_e_ of the pooled group of LBC and HBC individuals, error bars: 95% CI, vertical axis: 2(SE^2^_LBC_ + SE^2^_HBC_)^0.5^, horizontal axis: 2SE; Spearman rank correlation coefficient: *ρ* = 0.80, *n*_LBC_ = 230, *n*_HBC_ = 90, *n*_microsatellites_ = 17, *P*_randomization between body condition classes_ = 0.00034. Data are from southern Kruger. This figure shows that it was the microsatellites with low baseline PL-*H*_e_ (<0.56) that were associated with effects on body condition, i.e. a relatively large PL-*H*_e_ decrease in the LBC (low body condition) group relative to the HBC (high body condition) group. The positive correlation indicates that the HFCs (heterozygosity-fitness correlations) in southern Kruger can only be explained by LD between microsatellites and expressed genes.

The PL-*H*_e_ decrease in the LBC group from southern Kruger (relative to the HBC group) declined with increasing baseline PL-*H*_e_ (Spearman rank correlation coefficient (*ρ*) = 0.80; *P*_randomization_ = 0.00034, *n*_LBC_ = 230, *n*_HBC_ = 90; northern Kruger: *P*_randomization_ = 0.53, *n*_LBC_ = 68, *n*_HBC_ = 70; Figure 1). As discussed above, this correlation cannot be explained by genetic drift due to population stratification, which is expected to result in an opposite pattern, namely an increase in PL-*H*_e_ decline with increasing baseline PL-*H*_e_. The correlation seemed to occur independently of BTB status, considering that it was also significant among BTB-negative individuals from herds with a BTB prevalence lower than 10% (*ρ* = 0.72; *P*_randomization_ = 0.0039; *n*_herds_ = 6, *n*_LBC_ = 55, *n*_HBC_ = 38). Furthermore, the correlation was significantly stronger than the correlation in the null model, wherein allele frequencies were equalized between the LBC and HBC group in each herd (*P*_randomization per herd_ = 0.0057, *n*_herds_ = 17, *n*_LBC_ = 178, *n*_HBC_ = 90). Because inbreeding and genetic drift due to population stratification cannot explain the differences in PL-*H*_e_ between the LBC and the HBC group, the most likely explanation is that LD with expressed genes is the cause (local effects hypothesis).

### Majority alleles associated with low body condition

Appreciable PL-*H*_e_ decrease in the LBC group relative to the HBC group in southern Kruger occurred only among the eight microsatellites with a baseline PL-*H*_e_ lower than 0.56 (*BM3517*, *BM4028*, *ETH10*, *ETH225*, *INRA006*, *INRA128*, *TGLA227* and *TGLA263*) (Figure 1). Although a significant decrease in the average PL-*H*_e_ for these eight microsatellites (i.e. ML-*H*_e_ decrease) was only observed among males, the decrease was not significantly different between sexes (males: −0.086±0.046; *P*_randomization_ = 0.00056, *n*_LBC_ = 92, *n*_HBC_ = 42; females: −0.042±0.044; *P*_randomization_ = 0.076, *n*_LBC_ = 138, *n*_HBC_ = 48; difference between sexes: *P*_randomization per sex_ = 0.21; Table S2).

PL-*H*_e_ decrease among the eight microsatellites with low baseline PL-*H*_e_ (<0.56) was primarily due to alleles of highest frequency. Each of these microsatellites contained a majority allele with a frequency higher than 0.63 (range: 0.633–0.916), in contrast to the remaining nine microsatellites (range: 0.200–0.407). All eight majority alleles increased in frequency in the LBC group relative to the HBC group in southern Kruger (*P*_sign test_ = 0.0078, a sign test was used because of absence of significant LD; Figure S2). In contrast, seven out of nine alleles of highest frequency at the microsatellites with high baseline PL-*H*_e_ (>0.75) decreased in frequency (*P*_sign test_ = 0.18; Figure S2) in the LBC group relative to the HBC group.

**Figure 2:**
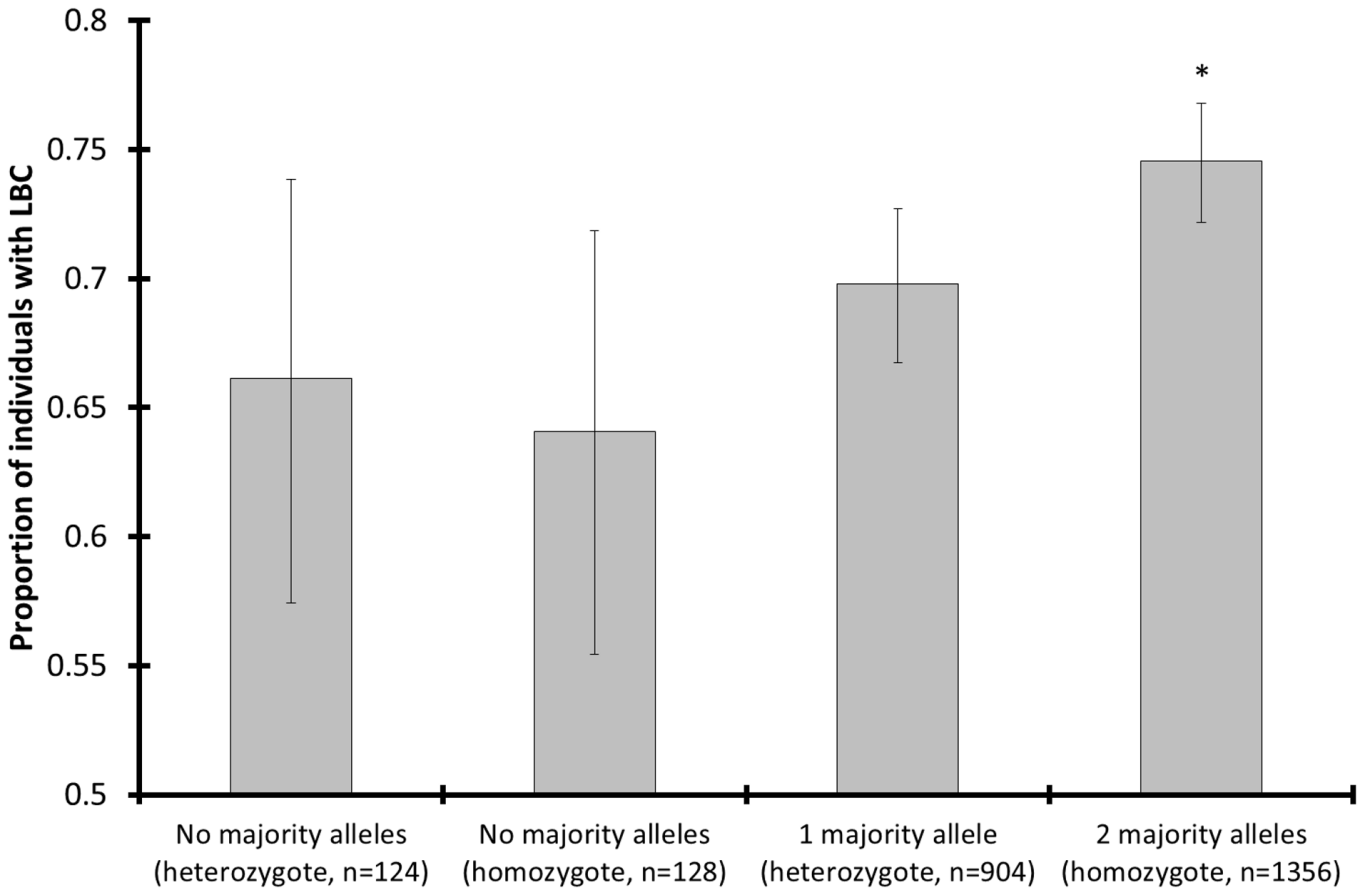
Proportion of LBC individuals for different classes of single-locus genotypes. Error bars: Wilson 95% CI, *: *P*_randomization_<0.033 compared to each of the other genotype classes. Data are from southern Kruger and only from those microsatellites that contained a majority allele. The high numbers of observations are due to the fact that the bars represent single-locus genotypes (eight times number of individuals). The single-locus genotypes could be pooled across microsatellites because there was no significant LD. This figure shows that only the homozygous majority alleles were associated with LBC (low body condition). The proportion of LBC individuals was significantly higher among majority-allele homozygotes than among any other class of single-locus genotype. Furthermore, the positive association between effect size and allele frequency (i.e. only significant effect with majority alleles) suggests that the majority alleles are linked to alleles at expressed genes under positive selection.

Among the single-locus genotypes at the microsatellites with low baseline PL-*H*_e_ (<0.56), the proportion of LBC individuals was significantly higher among majority-allele homozygotes than among any of the other genotype classes (χ^2^ tests; comparing all classes: χ^2^-value  = 12.65, *P*_randomization_ = 0.0053, *n*_genotypes_ = 2512; pairwise comparisons between majority-allele homozygotes and each of the other classes: *P*_randomization_<0.033; majority-allele homozygotes: 0.747±0.019, *n*_genotypes_ = 1356; other homozygotes: 0.629±0.093, *n*_genotypes_ = 128; heterozygotes with a majority allele: 0.698±0.030, *n*_genotypes_ = 904; heterozygotes without a majority allele: 0.661±0.082, *n*_genotypes_ = 124; Table S3–S6, Figure 2). Among the other genotype classes, no significant differences were observed in proportion of LBC individuals (χ^2^-value  = 2.17, *P*_randomization_ = 0.34, *n*_genotypes_ = 1156; Table S7). The difference in LBC proportion between majority-allele homozygotes on the one hand and all the other genotypes on the other was highly significant (χ^2^-value  = 10.34, *P*_randomization_ = 0.0013, *n*_genotypes_ = 2512; Table S8). It remained significant when compared against the null model (χ^2^-value summed across herds  = 3.68, *P*_randomization per herd_ = 0.021, *n*_genotypes_ = 2098, *n*_herds_ = 17), wherein allele frequencies were equalized between the LBC and HBC group in each herd. According to multiple logistic regression that included age and herd latitude, the total number of homozygous majority alleles carried by an individual from southern Kruger was negatively correlated with body condition (χ^2^-value  = 51.07, *P* = 0.0050, *n*_LBC_ = 230, *n*_HBC_ = 90, *n*_herds_ = 20; Table 1).

**Table 1:** Multiple logistic regression between an individual's body condition status and the number of homozygous majority alleles out of a possible eight.

Independent variable	Coefficient (B)	SE	Wald statistic	d.f.	*P-*value	Exponent of B
Number homozygous majority alleles	−0.289	0.103	7.878	1	5.0*10^−3^	0.749
Age (years)	−0.196	0.048	17.073	1	3.6*10^−5^	0.822
Latitude (degrees)	1.484	0.344	18.594	1	1.6*10^−5^	0.227
Constant	37.803	8.544	19.577	1	9.7*10^−6^	2.616*10^16^

Dependent variable: body condition: 0 =  LBC (low body condition), 1 =  HBC (high body condition). χ^2^-value model  = 51.07, d.f.  = 3, *P*_model_  = 4.7*10^−11^, *n*_LBC_ = 230, *n*_HBC_ = 90, *n*_herds_ 20. Sex (categorical variable) and BTB status (bovine tuberculosis, categorical variable) were not significant when added to the model (*P*>0.17). Forward and backward selection resulted in the same model.

The χ^2^ test and multiple logistic regression analysis indicate that it is predominantly the (homozygous) majority microsatellite alleles that were associated with deleterious alleles causing LBC, something one would not expect if the HFCs were caused by inbreeding (general effects hypothesis). This pattern, however, is consistent with the inference that the majority alleles are in LD with deleterious alleles at expressed genes (local effects hypothesis).

### Opposing allele frequency dynamics between sexes associated with body condition

In southern Kruger, the LBC-minus-HBC group difference in ML-*H*_e_ among the nine microsatellites with a baseline PL-*H*_e_ higher than 0.75 (*BM1824*, *BM3205*, *BM719*, *CSSM19*, *DIK020*, *ILSTS026*, *SPS115*, *TGLA159* and *TGLA57*) differed between females and males, which suggests sex-specific selection. Males showed a ML-*H*_e_ decrease in the LBC group relative to the HBC group, as was also observed with the eight microsatellites with a baseline PL-*H*_e_ lower than 0.56, although in this case the decrease was not significant (−0.017±0.019; *P*_randomization_ = 0.092, *n*_LBC_ = 92, *n*_HBC_ = 42; Table S2). However, females showed the opposite: a significant ML-*H*_e_ increase in the LBC group relative to the HBC group (+0.020±0.019; *P*_randomization_ = 0.021, *n*_LBC_ = 138, *n*_HBC_ = 48; Table S2). The LBC-minus-HBC difference in females was significantly different from that in males (*P*_randomization per sex_ = 0.0053; Table S2).

**Figure 3:**
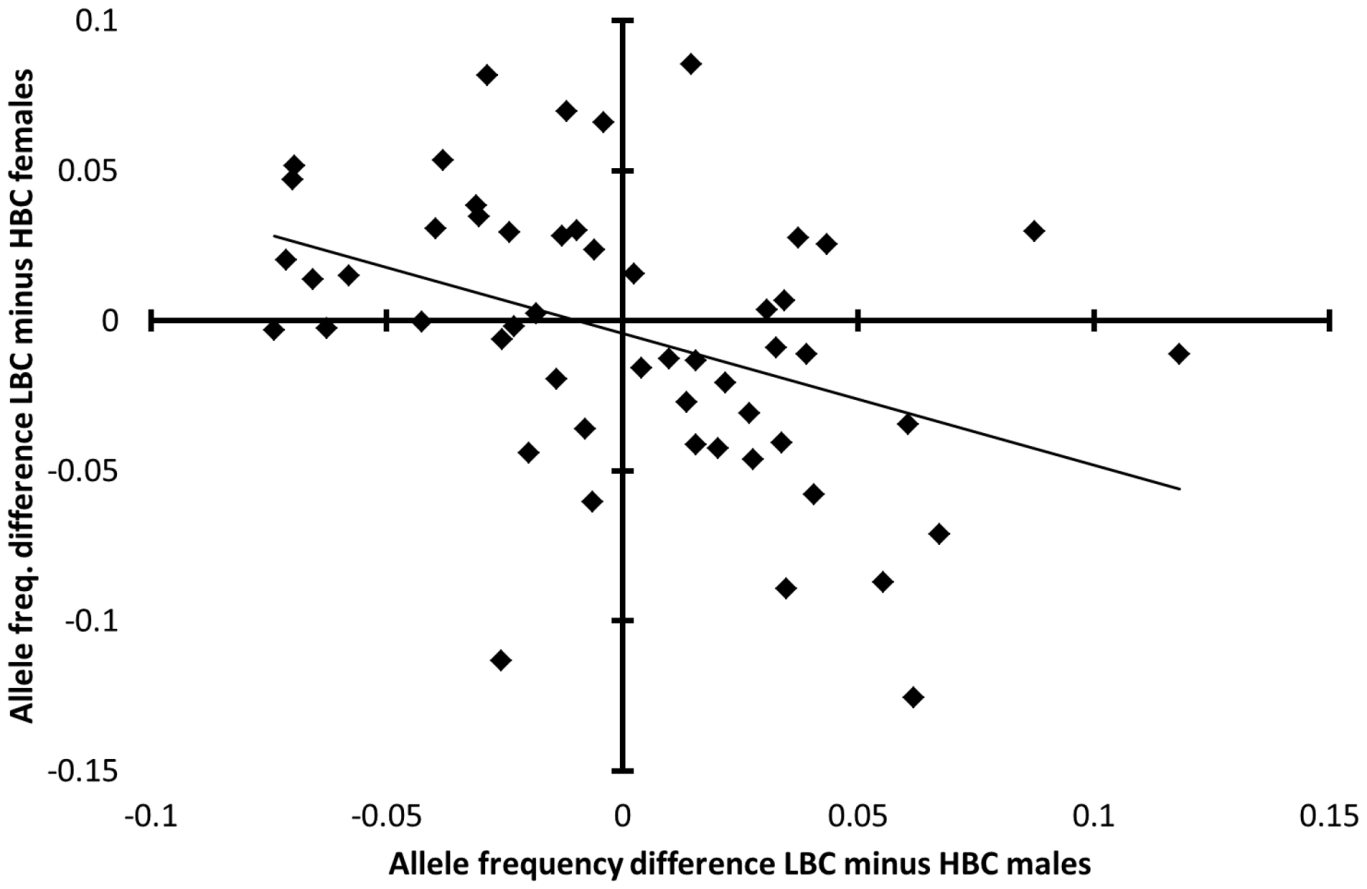
Negative correlation between males and females in LBC-minus-HBC allele frequency difference. Spearman rank correlation: *ρ* = −0.46, *n*_microsatellites_ = 9 (without majority allele), *n*_alleles_ = 53 excluding rare alleles (frequency <0.05) to prevent low sample size bias, *n*_LBC females_ = 138, *n*_HBC females_ = 48, *n*_LBC males_ = 92, *n*_HBC males_ = 42, *P*_randomization between body condition classes per sex_ = 0.0024. Data are from southern Kruger. The opposite sex effect (negative correlation), i.e. relatively high microsatellite allele frequencies in LBC (low body condition) males relative to HBC (high body condition) males and vice versa in females, indicates that the linked expressed gene alleles are sexually antagonistic.

The opposite-sex effect could be attributed to a significant negative correlation between LBC-minus-HBC allele frequency differences in males and those in females (LBC-minus-HBC difference males *vs.* LBC-minus-HBC difference females; *ρ* = −0.46, *P*_randomization per sex_ = 0.0024, *n*_LBC,females_ = 138, *n*_HBC,females_ = 48, *n*_LBC,males_ = 92, *n*_HBC,males_ = 42, *n*_microsatellites_ = 9, *n*_alleles_ = 53; northern Kruger: *P*_randomization per sex_ = 0.80, *n*_LBC,females_ = 46, *n*_HBC,females_ = 43, *n*_LBC,males_ = 22, *n*_HBC,males_ = 27, *n*_microsatellites_ = 9, *n*_alleles_ = 56; Figure 3). A negative correlation between sexes was observed at eight out of nine microsatellites (only TGLA159 showed a positive correlation). Alleles with a relatively high frequency in LBC compared to HBC males tended to have a relatively low frequency in LBC compared to HBC females. Further, the correlation was significantly more negative than in the null model, wherein allele frequencies were equalized between sexes and body condition classes in each herd (*P*_randomization per herd_ = 0.00094, *n*_herds_ = 20, *n*_individuals_ = 320), and also significantly more negative than in northern Kruger (*P*_randomization per herd_ = 0.012). Actually, the opposite pattern was observed in the null model; namely a positive correlation in LBC-minus-HBC allele frequency differences between sexes (Figure S3).

The opposing allele frequency dynamics between sexes indicate linkage to sexually antagonistic genes whose alleles, when expressed, have opposing effects on the body condition of males and females (we consider alternative explanations unlikely, see Text S1). The postulated sexually antagonistic alleles did not result in significant microsatellite allele frequency differences between sexes (population differentiation test, not assuming Hardy-Weinberg equilibrium: *P* = 0.16), suggesting only a limited effect on mortality.

**Figure 4:**
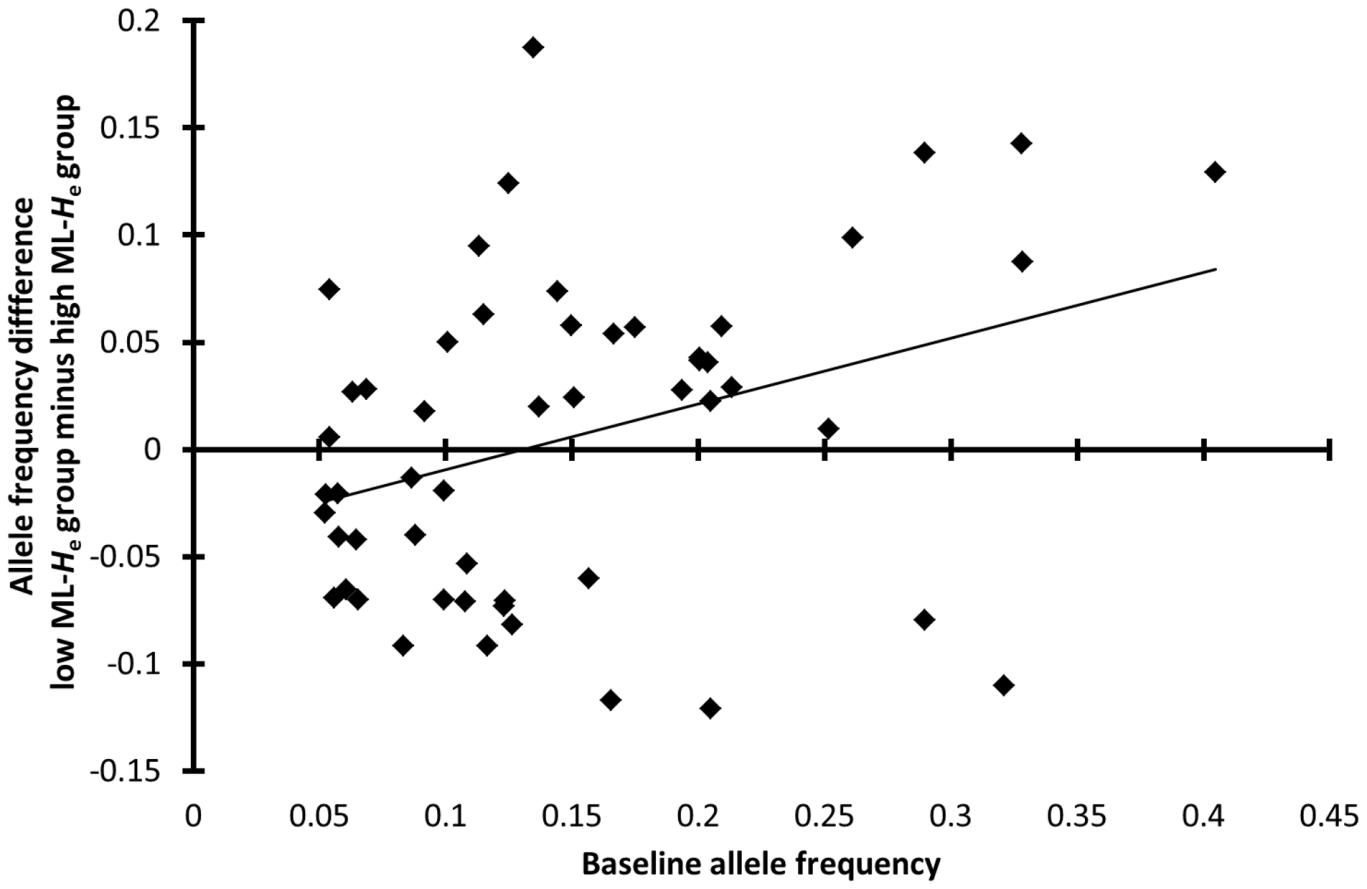
Allele frequency difference between low ML-*H*_e_ and high ML-*H*_e_ group correlated against baseline allele frequency. Low ML-*H*_e_ (multilocus expected heterozygosity) group: LBC males and HBC females (opposite body condition classes were combined because of sexual antagonism, see Figure 3), high ML-*H*_e_ group: HBC males and LBC females, baseline allele frequency: allele frequency among pooled LBC and HBC individuals of both sexes, Y-axis: allele frequencies were averaged across sexes, Spearman rank correlation coefficient: *ρ* = 0.32, *n*_microsatellites_ = 9 (without majority allele), *n*_alleles_ = 53 excluding rare alleles (frequency <0.05) to prevent low sample size bias, *n*_LBC males_ = 92, *n*_HBC males_ = 42, *n*_LBC females_ = 138, *n*_HBC females_ = 48, *P*_randomization between body condition classes per sex_ = 0.027. Data are from southern Kruger. The positive allele frequency differences at high baseline values indicate that most high-frequency alleles were associated with LBC (low body condition) in males and HBC (high body condition) in females. Furthermore, the positive correlation between effect size and baseline allele frequency suggests that most high-frequency alleles are linked to alleles at expressed genes under positive selection.

The allele frequency differences for both sexes combined, i.e. average allele frequency increase in LBC males and HBC females (low ML-*H*_e_ group) relative to HBC males and LBC females (high ML-*H*_e_ group) ( =  [LBC-minus-HBC difference males] minus [LBC-minus-HBC difference females]), increased with increasing baseline allele frequency (*ρ* = 0.32, *P*_randomization per sex_ = 0.027, *n*_LBC,females_ = 138, *n*_HBC,females_ = 48, *n*_LBC,males_ = 92, *n*_HBC,males_ = 42, *n*_microsatellites_ = 9, *n*_alleles_ = 53; Figure 4). This correlation was also observed when compared against the null model whereby allele frequencies were equalized between sexes and body condition classes in each herd (*P*_randomization per herd_ = 0.034, *n*_herds_ = 20, *n*_individuals_ = 320). So the largest effects, i.e. the sex-specific LBC-minus-HBC allele frequency differences, were observed for those alleles with the highest frequencies. The LBC-HBC allele frequency differences for both sexes combined were mostly positive at high baseline frequencies (21 out of 27 alleles with frequencies ≥0.125), which indicates that most of these alleles were associated with LBC in males and HBC in females rather than vice versa.

### Indications of positive selection

We estimate that the postulated deleterious alleles had a mean frequency of at least 0.029 (*P*_1-sided_ = 0.05) among LBC individuals from southern Kruger, considering the increase in the frequency of majority microsatellite alleles in LBC compared to HBC individuals (LBC: 0.735±0.016, HBC: 0.682±0.024; difference: 0.053±0.029, *n*_LBC_ = 230, *n*_HBC_ = 90). This estimate is about three times higher than the combined frequency of deleterious alleles for an average expressed gene in humans, which is estimated at 0.01 [34]. High frequencies of deleterious alleles together with their strong association with majority microsatellite alleles suggest positive rather the anticipated negative selection [35]. Majority microsatellite alleles may have attained their current high frequency because of positive selection across multiple generations.

Also with the microsatellite alleles linked to sexually-antagonistic alleles the largest effects were observed for those with the highest baseline frequencies (Figure 4), which again indicates positive selection. Those microsatellite alleles with the largest sex-specific LBC-minus-HBC differences are expected to be linked to sexually antagonistic alleles under the strongest positive selection and therefore show the highest frequencies after multiple generations of selection.

**Figure 5:**
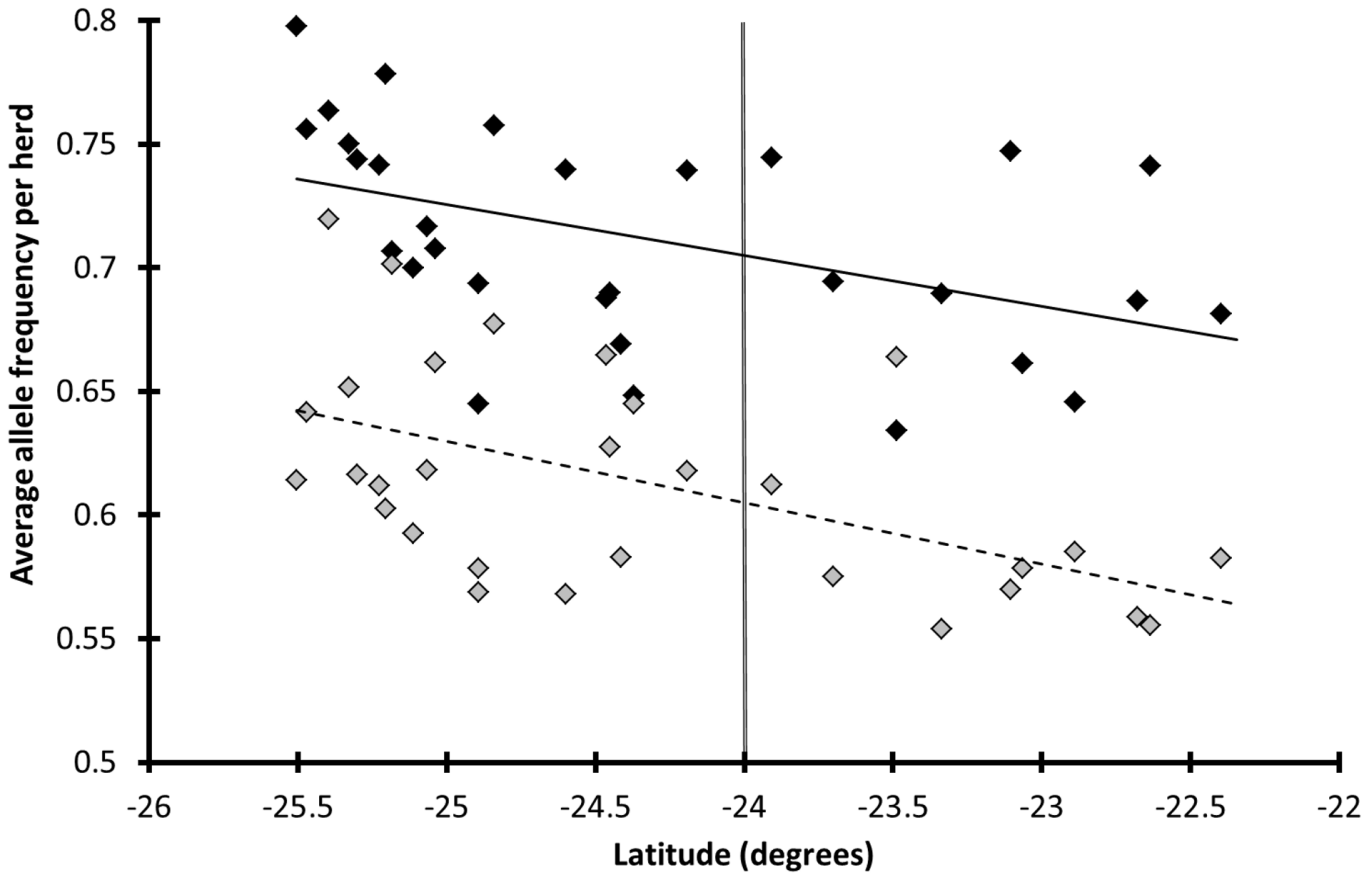
Allele clines in Kruger for two classes of microsatellites. Allele frequencies were averaged across individuals and across microsatellites. Black diamonds: average majority allele frequency per herd, *Z* = 3.51, *P*_Stouffer-*Z*_ = 0.00044, *n*_microsatellites_ = 8, *ρ* = −0.60, *n*_herds_ = 30, *n*_individuals_ = 459; grey diamonds: average frequency per herd of the pooled three most frequent alleles per microsatellite (microsatellites without majority allele), *Z* = 3.58, *P*_Stouffer-*Z*_ = 0.00034, *n*_microsatellites_ = 9, *ρ* = −0.52, *n*_herds_ = 30, *n*_individuals_ = 459, latitude <−24: southern Kruger. Allele clines with increasing frequencies going from north to south were observed for all eight autosomal microsatellites with a majority allele and eight out of nine autosomal microsatellites without a majority allele. These allele clines are indicative of positive selection.

Positive selection of deleterious and sexually antagonistic alleles was strongly supported by the observation of an allele cline at 16 of the 17 microsatellites, with frequencies increasing in a north-south direction. Allele clines are generally considered as a strong indicator of positive selection [36]–[38]. All eight majority microsatellite alleles showed a negative Spearman rank correlation with latitude (*Z* = 3.51, *P*_Stouffer-*Z*_ = 0.00044, *n*_microsatellites_ = 8; Table S9, Figure 5). They increased in frequency from around 0.67 in the far north to around 0.74 in the far south. Allele clines were also observed at the remaining microsatellites (baseline PL-*H*_e_ >0.75) when the three most frequent alleles per locus were pooled. This pooling assumes that these frequent alleles, considering the relatively large positive effect size for most of them (21 out of 27 alleles with frequencies ≥0.125 showed a positive effect size; Figure 4), are much more likely to be linked to a sexually antagonistic allele than the remaining less frequent alleles. Because of this linkage, these microsatellite alleles may to a large extent behave as a single allele. Indeed, eight of the nine pooled alleles showed a negative Spearman rank correlation with latitude (*Z* = 3.58, *P*_Stouffer-*Z*_ = 0.00034, *n*_microsatellites_ = 9; Table S10, Figure 5). The significance of the latter result cannot be explained by a negative bias on the *P*-value because of LD among three of the microsatellites, considering that the correlation between latitude and the ‘average allele frequency across loci’ (after pooling within loci) was also highly significant (*ρ* = −0.52, *P* = 0.0030, *n*_herds_ = 30). The pooled alleles increased in frequency from around 0.56 in the far north to around 0.64 in the far south.

### Statistical associations between autosomal and Y-chromosomal microsatellites

Positive selection of microsatellite alleles associated with LBC very likely implies that on average LBC individuals, or a certain group of LBC individuals, had a higher relative fitness than HBC individuals. This higher relative fitness is most likely related to increased fertility, because it is highly unlikely that LBC by itself can increase lifetime mating success.

We hypothesized that an increased fertility among LBC individuals is related to sex-ratio distorter and suppressor genes. The occurrence of these genes in the Kruger buffalo population has been suggested in a previous study using Y-chromosomal microsatellites. Sex-ratio distorters generally have a negative effect on fertility [13]. Sex-ratio distorters and suppressors may have a different effect on LBC individuals than on HBC individuals, especially considering that the activity of these genes in the Kruger buffalo population has been associated with body condition [13]. If Y-chromosomal sex-ratio distorters and suppressors were involved, then one expects associations between the Y-chromosomal and the autosomal microsatellite data, which was indeed observed.

**Figure 6:**
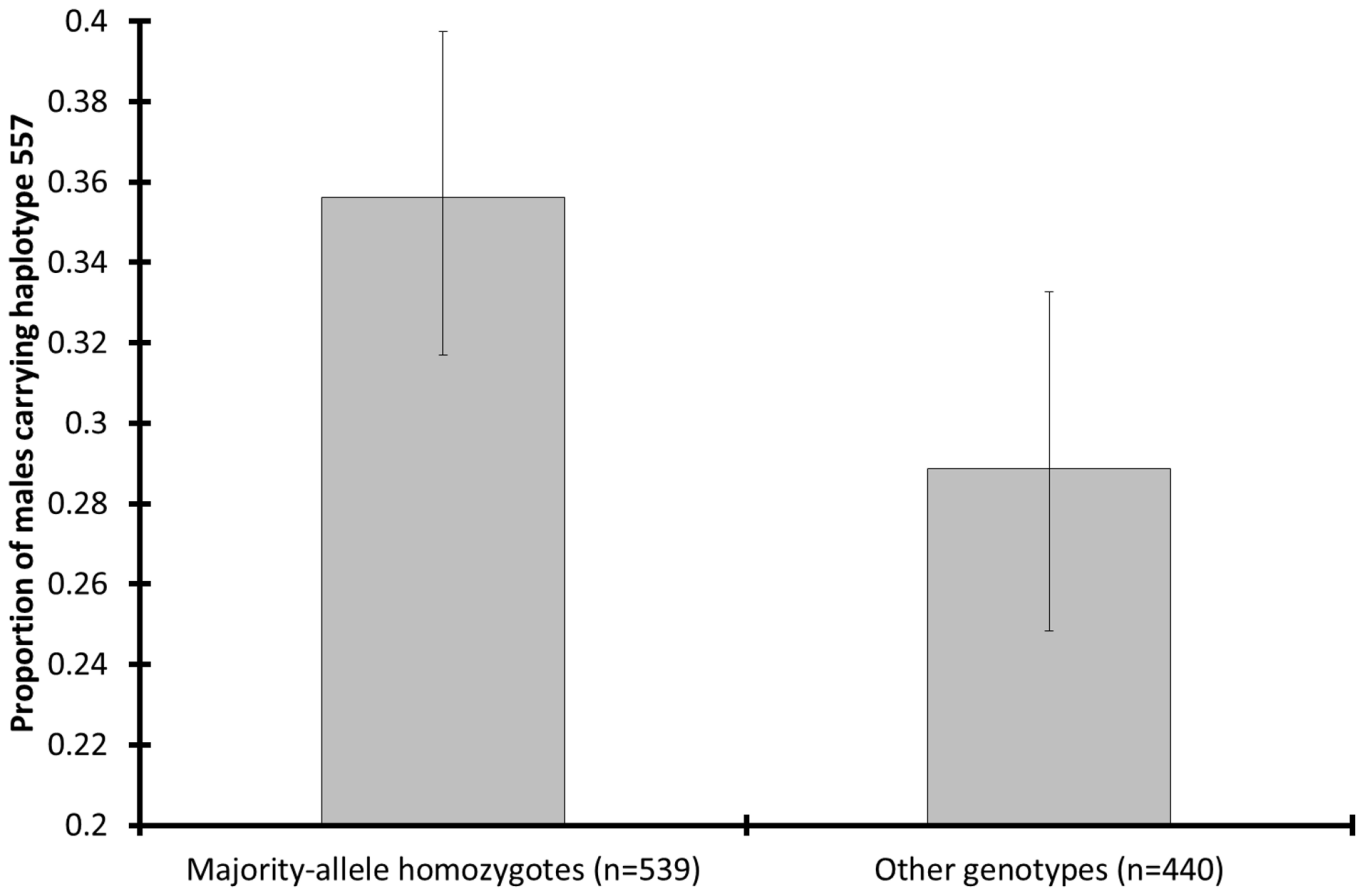
Proportion of males carrying Y-chromosomal haplotype 557 in relation to autosomal genotype. Error bars: Wilson 95% CI. Data are from southern Kruger and from only those microsatellites that contained a majority allele. The high numbers of observations are due to the fact that the bars represent single-locus genotypes (eight times number of individuals). The single-locus genotypes could be pooled across microsatellites because there was no significant LD. This figure shows a direct association at the individual level between autosomal deleterious alleles (in LD with homozygous microsatellite majority alleles) and a Y-chromosomal sex-ratio suppressor (in LD with haplotype 557). The proportion of males carrying haplotype 557 was significantly higher among majority-allele homozygotes than among the other single-locus genotypes (*P*_randomization_ = 0.019).

Homozygous majority alleles were relatively common among 557-carrying males in southern Kruger (proportion 557-carrying males among single-locus majority-allele homozygotes: 0.36±0.04, proportion 557-carrying males among other single-locus genotypes: 0.29±0.04; χ^2^-value  = 5.04, *P*_randomization_ = 0.019, *n*_genotypes_ = 979; Table S11, Figure 6). The result remained significant when compared against the null model, wherein allele frequencies were equalized between 557-carrying males and other males in each herd (χ^2^-value summed across herds  = 3.37, *P*_randomization per herd_ = 0.034, *n*_genotypes_ = 823, *n*_herds_ = 18).

Two indirect but highly significant associations (*P*≤0.0014) between the Y-chromosomal and autosomal microsatellite data were also observed: 1) both type of markers showed allele clines running in a north-south direction (Figure S4) and 2) both ML-*H*_e_ per year-cohort and frequency of haplotype 557 per year-cohort were significantly correlated with preconception rainfall (Figure S5). In a previous study it was observed that the year-cohort frequencies of haplotype 557 correlated with rainfall that occurs for a period prior to conception, with the strongest correlations for a three-year preconception rainfall period [13]. This correlation is likely due to differentials in the fertility of 557-carrrying males in relation to body condition after extended wet and dry periods lasting multiple years [13]. Details of the indirect associations are provided with Figures S4 and S5.

## Discussion

### Unusual genetic processes in the Kruger buffalo

This study contains several novel observations on HFCs not reported elsewhere. These include: 1) HFCs with the great majority of the studied microsatellites, 2) a fitness parameter (here body condition) strongly associated with a particular class of microsatellite alleles (majority alleles), 3) opposing allele frequency dynamics between sexes, 4) associations between allele frequency and effect on fitness (our surrogate measure is body condition), 5) the occurrence of allele clines and 6) associations between autosomal and Y-chromosomal microsatellite data. Such a wide range of distinctive observations suggests that unusual genetic processes are occurring in the Kruger buffalo. Various traditional explanations were considered by us, such as null alleles, sample size variation, sex-biased dispersal, sex-specific age distribution, involvement of fertility-enhancing alleles and recent population bottlenecks, but all failed to explain the entire range of previously-mentioned observations (Text S1).

### Genome-wide positive selection of alleles deleterious to male body condition driven by a Y-chromosomal sex-ratio suppressor

We hypothesize that the results we obtained from our analyses of the autosomal microsatellite data are due to positive selection of alleles at linked genes that are deleterious to the body condition of males. Positive selection implies that these alleles increase the relative fitness of their carriers, despite their negative effect on body condition. This positive selection has resulted in elevated frequencies of deleterious alleles, deleterious to both sexes (relatively weakly deleterious in females), and sexually antagonistic alleles, deleterious to males only. Positive selection seems to occur genome-wide, considering that the great majority of microsatellites, which were randomly chosen with respect to genomic location, showed signs of LD with an expressed gene. Hundreds of autosomal genes must be involved with selection when we extrapolate from the small set of microsatellites that we used.

Results from a previous study suggest that Y-chromosomal haplotype 557 is in LD with a sex-ratio suppressor [13]. We therefore hypothesize that the observed associations between autosomal and Y-chromosomal microsatellite data are a result of positive selection, with the selection agent being a Y-chromosomal sex-ratio suppressor in males that is activated by low body condition (LBC). Haplotype 557 being activated by LBC is suggested, on the one hand, by the association of Y-chromosomal haplotype 557 with number of homozygous majority alleles, and on the other hand, by the association of homozygous majority alleles with LBC.

**Figure 7:**
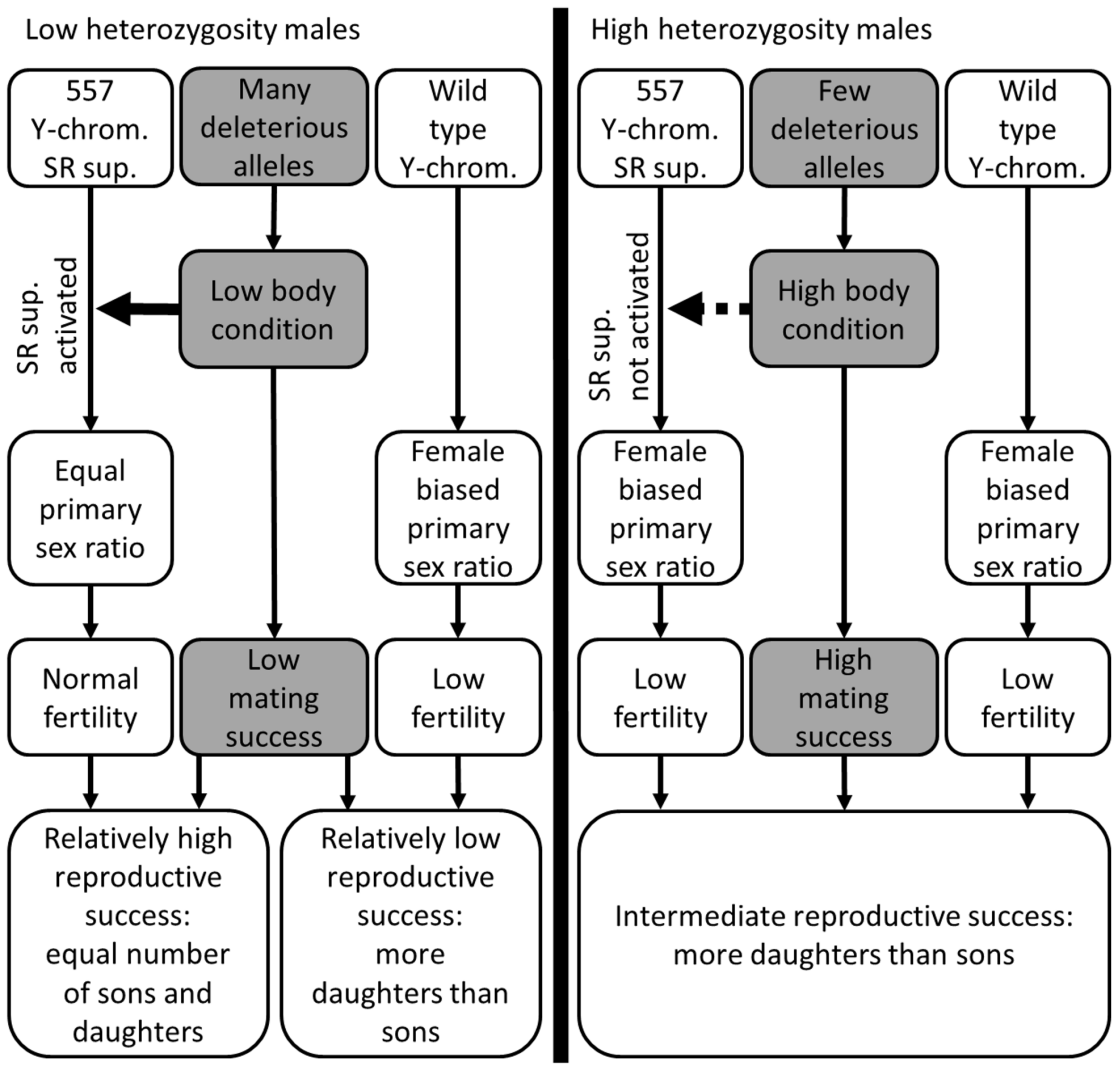
A hypothesised mechanism that can explain positive selection of alleles deleterious to male body condition. A sex-ratio (SR) suppressor, linked to Y-chromosomal haplotype 557, is triggered by low body condition. When active, it prevents a decrease in fertility that is due to SR distortion. Low body condition can be caused by a lack of resources during droughts and by disease. A SR distorter, resulting in a female-biased sex ratio [13], is assumed to be active in a large fraction of males. Body-condition associated suppressor activity can result in positive selection of deleterious alleles that negatively affect male body condition, provided that the positive effect on relative male fertility is larger than the negative effect on lifetime male mating success and that their effect on female relative fitness is not too negative. Thus there is a trade-off between increased relative fertility and decreased lifetime mating success. Positive selection can occur as long as the net reproductive success is higher for low-heterozygosity males than for high-heterozygosity males. The net reproductive success of low-heterozygosity males is positively correlated with the SR suppressor frequency (relatively high in southern Kruger, Figure S4) and with the negative effect of the SR distorter on male fertility. The latter effect is probably large considering the strong frequency fluctuations (up to a factor of five) of the SR suppressor between wet periods, when it is not active, and dry periods, when it is active [13].

Relatively high frequencies of haplotype 557 after dry years, as observed in a previous study, suggest that the sex-ratio suppressor gives its carrier a reproductive advantage when its body condition is low [13]. This increased relative reproductive success is best explained by assuming that the sex-ratio suppressor is activated by LBC, thereby suppressing a female-biased sex-ratio distortion (Figure 7). Female-biased foetal sex ratios have been observed in the Kruger buffalo population [13]. This suppression gives LBC 557-carrying males a relative fertility advantage compared to the other males (LBC males without haplotype 557 and HBC males irrespective of Y-chromosomal haplotype), which suffer from fertility reduction due to (unsuppressed) sex-ratio distortion. Any suppressor is expected to have a selective advantage relative to the non-suppressing wild-type, even when only active in LBC individuals [13]. As a result, any deleterious allele that lowers male body condition, as long as lowered body condition does not compromise lifetime mating success too much, may give a fitness advantage to sex-ratio suppressor carriers, resulting in genome-wide positive selection (provided the effect on female relative fitness is not too negative). In other words, positive selection of deleterious alleles is possible because there is a trade-off between increase in relative fertility and decrease in lifetime mating success. This trade-off occurs not within individuals, but among individuals at population scale.

The trade-off between increase in relative fertility and decrease in lifetime mating success occurs only among males, because sex-ratio genes primarily affect the proportion of viable X- and Y-spermatozoa, which would explain the failure to observe sexually antagonistic alleles that are beneficial to the body condition of males and why the postulated deleterious alleles had a relatively strong effect on male body condition (although the difference with females was not significant). Furthermore, the mechanism outlined above also explains why haplotype 557 was associated with homozygous majority microsatellite alleles. Individual males with a relatively high number of homozygous majority alleles are expected to produce most of the offspring among males with haplotype 557 because of high relative fertility due to an active sex-ratio suppressor, but least of the offspring among males without haplotype 557 because of low mating success due to low body condition. As a result the frequency of homozygous majority alleles increases in the offspring of 557-carrying males, but decreases in the offspring of other males.

### Abundance of sexually antagonistic genes in the buffalo genome

The observation that the majority of microsatellites with a baseline PL-*H*_e_ larger than 0.75 seemed to be in LD with a sexually antagonistic gene suggests that sexually antagonistic genes are abundant in the African buffalo genome [39]. The fraction of genes in the African buffalo with sexually antagonistic properties may be comparable to the estimate of 8% obtained in *Drosophila melanogaster* [40]. However, this latter estimate is from a laboratory population that may have skewed results [40], [41]. Our study is the first to provide an estimate of the abundance of sexually antagonistic genes in a natural population. Thereby, our study provides strong support to the view that intra-locus sexual conflict may be a fundamental factor for the genetic architecture of fitness and may be important for conserving genetic polymorphism [42], [43].

### A recent selective sweep

The association of deleterious alleles with majority microsatellite alleles indicates high population frequencies of the former. The majority alleles from the five microsatellites that were also used in a previous study on African buffalo (*BM3517*, *BM4028*, *INRA006*, *INRA128* and *TGLA263*), have considerably higher frequencies in Kruger than in East Africa, suggesting deleterious allele frequencies of at least 0.2 (frequency Kruger  = 0.690 (*n* = 459), frequency East Africa  = 0.473 (*n* = 110); raw data from [44], http://doi.org/10.5061/dryad.23d13). This high frequency estimate and the seemingly tight association with a single microsatellite allele (i.e. majority allele) suggest a high selection coefficient in the recent evolutionary past.

Apparently, the frequency increase across generations of linked allele pairs consisting of deleterious alleles and majority microsatellite alleles occurred much faster than LD decay of these linked allele pairs due to recombination. The absence of significant LD decay suggests that the sex-ratio suppressor, i.e. the selective agent, evolved relatively recently. However, a fast frequency increase only seems possible when the deleterious alleles are additive or dominant rather than recessive. New mutations that are additive or dominant more readily spread through a population than those that are recessive, because new mutations are initially rare in a population and homozygotes for a new mutation even more so (square of initial allele frequency). They are thus easily lost by genetic drift, even when they are highly beneficial (Haldane's sieve: the bias against the establishment of recessive mutations [45]). The deleterious alleles in this study could well be additive, considering that the proportion of LBC individuals among single-locus genotypes with one majority allele (heterozygotes) was precisely in-between that among single-locus genotypes with no majority allele and single-locus genotypes with two majority alleles (proportion of LBC individuals per genotype class: majority-allele homozygotes: 0.75, majority- allele heterozygotes: 0.70, other genotypes: 0.65; Figure 2).

Microsatellites alleles appeared to be less strongly linked to sexually antagonistic alleles than to deleterious alleles. This indicates a relatively large physical distance between sexually antagonistic genes and microsatellites or a relatively weak effect of selection, possibly due to a stronger selective effect of nearby deleterious alleles or constraints in sex differences of sexually antagonistic phenotypic traits [46]. Alternatively, the sexually antagonistic alleles may not constitute new mutations (on an evolutionary timescale), but reflect a more ancient polymorphism being maintained in the population by balancing selection (balancing selection due to opposite fitness effects in the two sexes) [43].

### Ecological effects and environmental interactions

Our results indicate that increased genetic load due to genome-wide positive selection of deleterious alleles has a considerable negative impact on population viability. LBC is known to increase susceptibility to and intensity of infection, not only of BTB but of most other diseases as well [47]. The higher BTB prevalence in southern than in northern Kruger, though initially due to a southern source for the infection [9], may now, in part, be a consequence of the effect of deleterious alleles on body condition.

The effects of deleterious and sexually antagonistic alleles on male and female body condition seemed to be conditionally expressed, considering that significant results were only observed in southern Kruger (a result unlikely to be attributed to the relatively small sample size in northern Kruger, see Text S1). Apparently, the deleterious and sexually antagonistic alleles were not or only weakly expressed, at least at the moment of sampling, in northern Kruger. Conditional expression of deleterious alleles is generally also observed with inbreeding depression [48]. Here the expression of deleterious and sexually antagonistic alleles probably interacted with spatiotemporal variation in environmental conditions, since the genetic backgrounds were very similar in northern and southern Kruger (*F*_ST_ = 0.005±0.002).

We do not know which environmental factors are at play in southern Kruger in the activation of alleles that affect body condition. These environmental factors seem to activate a wide range of diverse genes, considering that body condition is a typical multi-gene trait involving complex multifactorial characters [49] and the genome-wide involvement of both sexually antagonistic and sex-independent genes. Activation of a wide range of diverse genes may occur via epigenetic interactions [50], [51] with one or a few master regulators of gene expression [52]. The fact that the geographic area of significant genetic effects on body condition closely corresponded with that of high BTB prevalence (i.e. southern Kruger) suggests that BTB may play a role in gene activation. However, if so, it must be an indirect role, for example through epigenetic inheritance, in order to explain the occurrence of HFCs in BTB-negative individuals.

## Conclusions

Our study shows that the African buffalo of Kruger National Park are undergoing a major genome-wide selective sweep of alleles deleterious to males, which we hypothesize is driven by a complex, newly discovered positive selection mechanism. Elevated frequencies of deleterious and sexually agonistic alleles in its genome makes the African buffalo an ideal model species for future genomic studies. These studies may provide important new insights into a wide range of population genetic, evolutionary and ecological fields: the functioning of deleterious and sexually agonistic alleles in mammals, Bovidae and cattle in particular, their interaction with environmental conditions, the evolutionary and ecological dynamics of sex-ratio distorters and suppressors, and the influence of genetic load on disease prevalence and population viability.

## Supporting Information

Figure S1 **Map of Kruger National Park with localities of the sampled herds.**
(DOCX)

Figure S2 **Differences in frequency of alleles of highest frequency between LBC and HBC individuals (one per locus).**
(DOCX)

Figure S3 **Positive correlation between males and females in LBC-minus-HBC allele frequency difference in the null model (Spearman rank correlation:** ***ρ*** **= 0.83).**
(DOCX)

Figure S4 **Cline of Y-chromosomal haplotype 557.**
(DOCX)

Figure S5 **Regression between ML-** ***H*** **_e_ per year-cohort and preconception rainfall.**
(DOCX)

Table S1 **Summary of the** ***P*** **-values in statistical tests for associations between body condition status and various genetic parameters.**
(DOCX)

Table S2 **ML-** ***H*** **_e_ per sex, body condition class and type of microsatellite (with or without a majority allele).**
(DOCX)

Table S3 ***Χ*** **^2^-test for differences in frequency distribution of single-locus genotypes between LBC and HBC individuals from southern Kruger (all genotype classes; baseline PL-** ***H*** **_e_ <0.56).**
(DOCX)

Table S4 ***Χ*** **^2^-test for differences in frequency distribution of single-locus genotypes between LBC and HBC individuals from southern Kruger (homozygotes with vs. homozygotes without a majority allele; baseline PL-** ***H*** **_e_ <0.56).**
(DOCX)

Table S5 ***Χ*** **^2^-test for differences in frequency distribution of single-locus genotypes between LBC and HBC individuals from southern Kruger (homozygotes with a majority allele vs. heterozygotes without a majority allele; baseline PL-** ***H*** **_e_ <0.56).**
(DOCX)

Table S6 ***Χ*** **^2^-test for differences in frequency distribution of single-locus genotypes between LBC and HBC individuals from southern Kruger (homozygotes vs. heterozygotes with a majority allele; baseline PL-** ***H*** **_e_ <0.56).**
(DOCX)

Table S7 ***Χ*** **^2^-test for differences in frequency distribution of single-locus genotypes between LBC and HBC individuals from southern Kruger (all genotype classes, excluding homozygotes with a majority allele; baseline PL-** ***H*** **_e_ <0.56).**
(DOCX)

Table S8 ***Χ*** **^2^-test for differences in frequency distribution of single-locus genotypes between LBC and HBC individuals from southern Kruger (homozygotes with a majority allele vs. remaining genotypes without a homozygous majority allele pooled; baseline PL-** ***H*** **_e_ <0.56).**
(DOCX)

Table S9 **Stouffer** ***Z*** **-test combining** ***P*** **-values of Spearman rank correlation per locus (correlation between allele frequency and latitude; baseline PL-** ***H*** **_e_ <0.56).**
(DOCX)

Table S10 **Stouffer** ***Z*** **-test combining** ***P*** **-values of Spearman rank correlation per locus (correlation between total frequency of the three most frequent alleles per locus and latitude; baseline PL-** ***H*** **_e_ >0.75).**
(DOCX)

Table S11 ***Χ*** **^2^-test for difference in frequency distribution of homozygous majority alleles between males with and males without haplotype 557 (baseline PL-** ***H*** **_e_ <0.56).**
(DOCX)

Text S1 **Alternative explanations.**
(DOCX)
