# Supplementary material for: Positive Selection of Deleterious Alleles through Interaction with a Sex-Ratio Suppressor Gene in African Buffalo: A Plausible New Mechanism for a High Frequency Anomaly
Source: PLoS One. 2014 Nov 5;9(11):e111778. doi: 10.1371/journal.pone.0111778 (PMC4221135; doi:10.1371/journal.pone.0111778)
Supplement: Figure S1 — Map of Kruger National Park with localities of the sampled herds. (DOCX) [file pone.0111778.s001.docx]

**
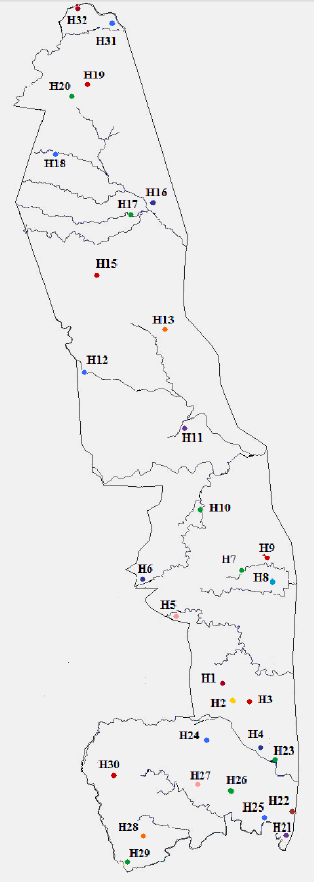
**

**Figure S1**: Map of Kruger National Park with localities of the sampled herds

Lines within park boundaries denote rivers with the horizontal one in the middle being the Olifants River, which delineates the northern and southern section. Numbers denote herd ID. The distance between the two herds farthest apart (herd 29 and herd 32) is 352 km. Data on physiology (disease status, age, body condition and sex), autosomal DNA and Y-chromosomal DNA were available for each herd, except herd 32, for which only data on physiology and Y-chromosomal DNA were available, and herd 13, for which only data on physiology were available. Figure 2.2 in Greyling BJ (2007) Genetic variation, structure and dispersal among Cape buffalo populations from the Hluhluwe-Imfolozi and Kruger National Parks of South Africa [PhD thesis]. Pretoria: University of Pretoria. 205 p.
