# Supplementary material for: Positive Selection of Deleterious Alleles through Interaction with a Sex-Ratio Suppressor Gene in African Buffalo: A Plausible New Mechanism for a High Frequency Anomaly
Source: PLoS One. 2014 Nov 5;9(11):e111778. doi: 10.1371/journal.pone.0111778 (PMC4221135; doi:10.1371/journal.pone.0111778)
Supplement: Figure S2 — Differences in frequency of alleles of highest frequency between LBC and HBC individuals (one per locus). (DOCX) [file pone.0111778.s002.docx]

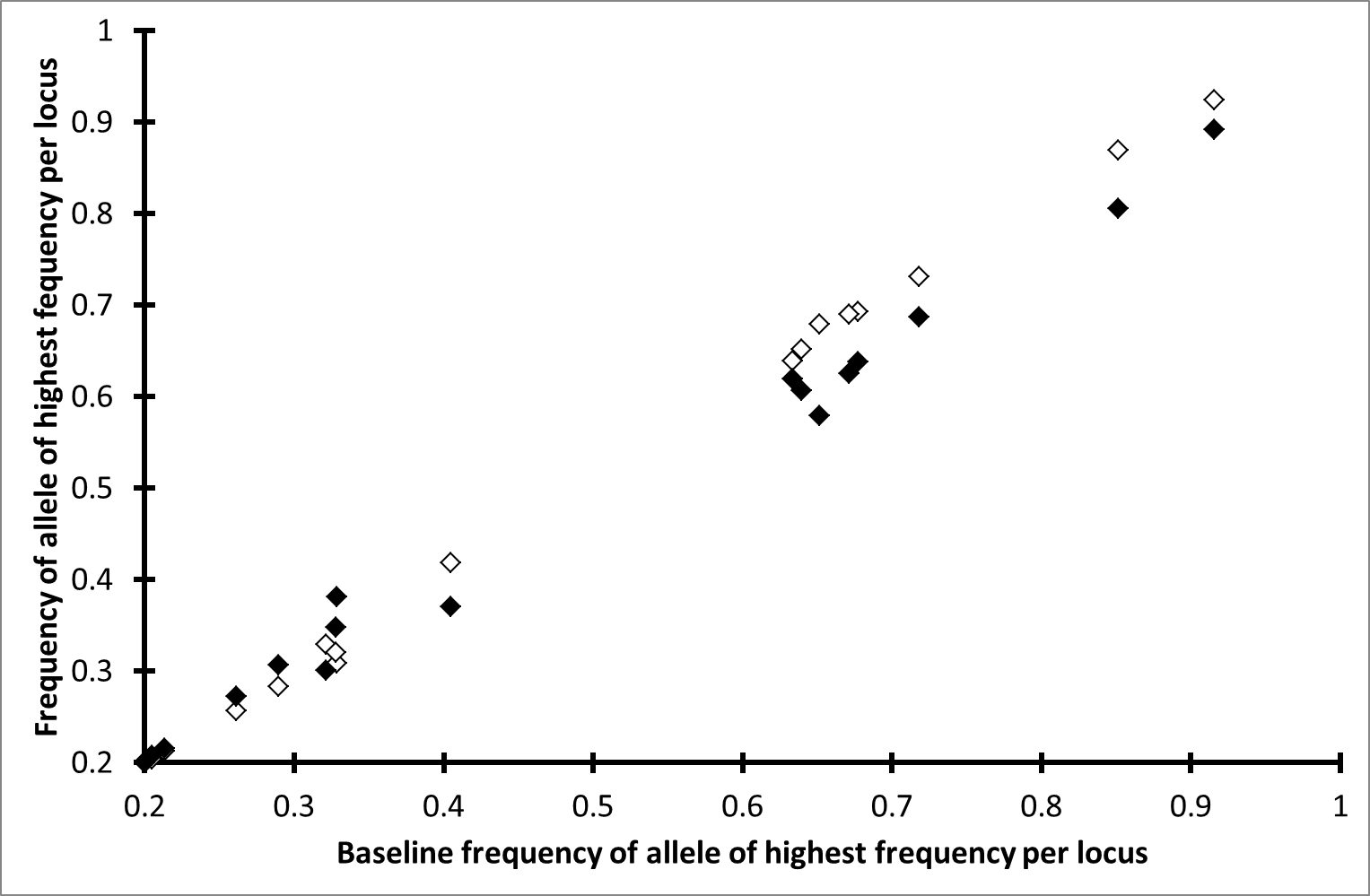


**Figure S2**: Differences in frequency of alleles of highest frequency between LBC and HBC individuals (one per locus)

White diamonds: low body condition (LBC), black diamonds: high body condition (HBC). *n*_LBC_ = 230, *n*_HBC_ = 90, *n*_microsatellites_ = 17. Data are from southern Kruger.
