# Supplementary material for: Positive Selection of Deleterious Alleles through Interaction with a Sex-Ratio Suppressor Gene in African Buffalo: A Plausible New Mechanism for a High Frequency Anomaly
Source: PLoS One. 2014 Nov 5;9(11):e111778. doi: 10.1371/journal.pone.0111778 (PMC4221135; doi:10.1371/journal.pone.0111778)
Supplement: Figure S3 — Positive correlation between males and females in LBC-minus-HBC allele frequency difference in the null model (Spearman rank correlation: ρ = 0.83). (DOCX) [file pone.0111778.s003.docx]

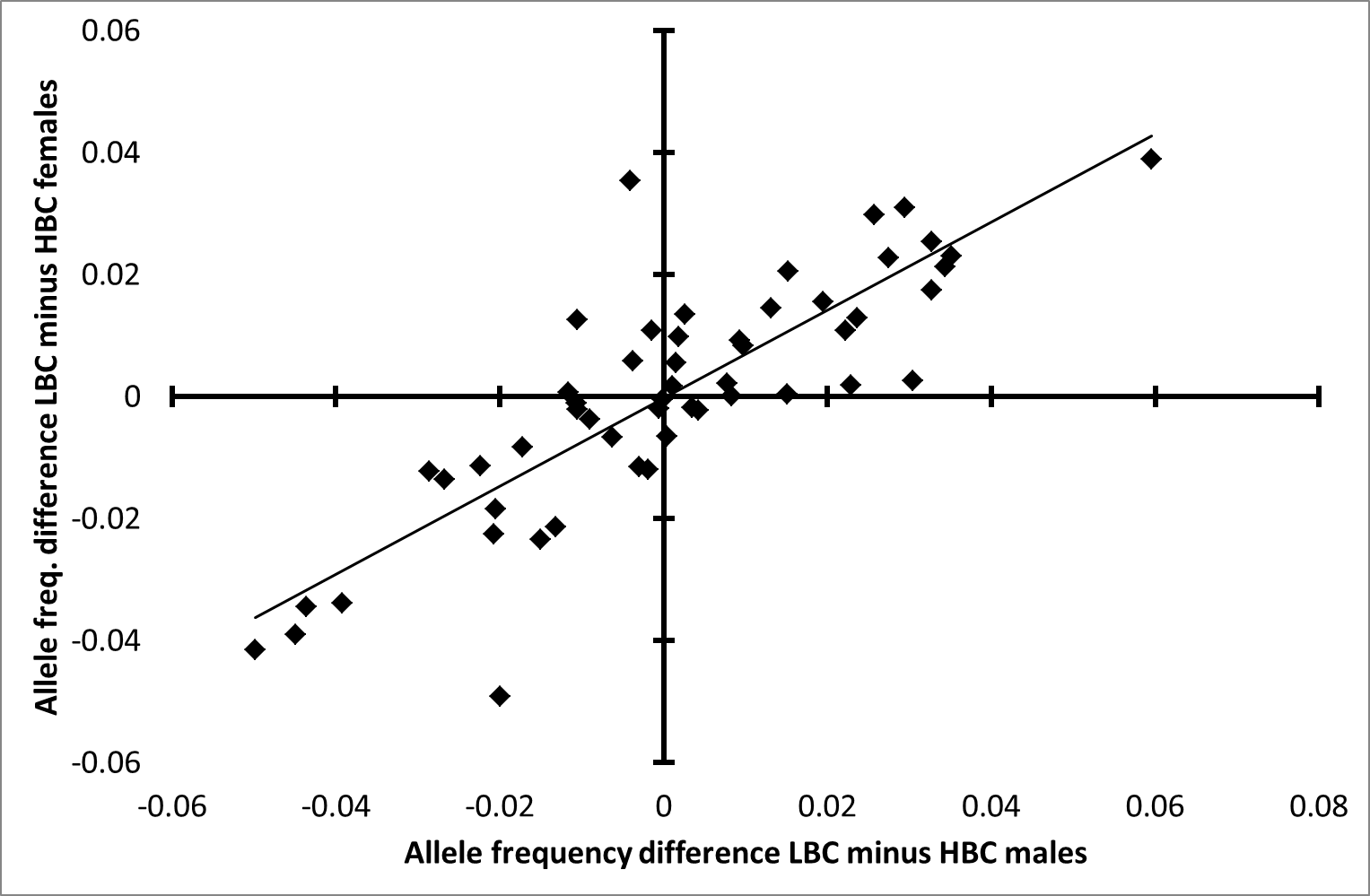


**Figure S3**: Positive correlation between males and females in LBC-minus-HBC allele frequency difference in the null model (Spearman rank correlation: *ρ* = 0.83)

*n*_alleles_ = 53 excluding rare alleles (frequency < 0.05) to prevent low sample size bias, *n*_LBC females_ = 138, *n*_HBC females_= 48, *n*_LBC males_ = 92, *n*_HBC males_ = 42, *n*_herds_ = 20. Data are from southern Kruger. In the null model allele frequencies were equalized between sexes and body condition classes in each herd. Expected counts of alleles per herd were subsequently summed across herds. The allele frequencies depicted in the figure are based on these summed allele counts. A positive correlation indicates that both allele frequencies per herd and proportion of LBC individuals per herd were correlated between sexes. Abbreviations: LBC: low body condition, HBC: high body condition.
