# Supplementary material for: Positive Selection of Deleterious Alleles through Interaction with a Sex-Ratio Suppressor Gene in African Buffalo: A Plausible New Mechanism for a High Frequency Anomaly
Source: PLoS One. 2014 Nov 5;9(11):e111778. doi: 10.1371/journal.pone.0111778 (PMC4221135; doi:10.1371/journal.pone.0111778)
Supplement: Figure S4 — Cline of Y-chromosomal haplotype 557. (DOCX) [file pone.0111778.s004.docx]

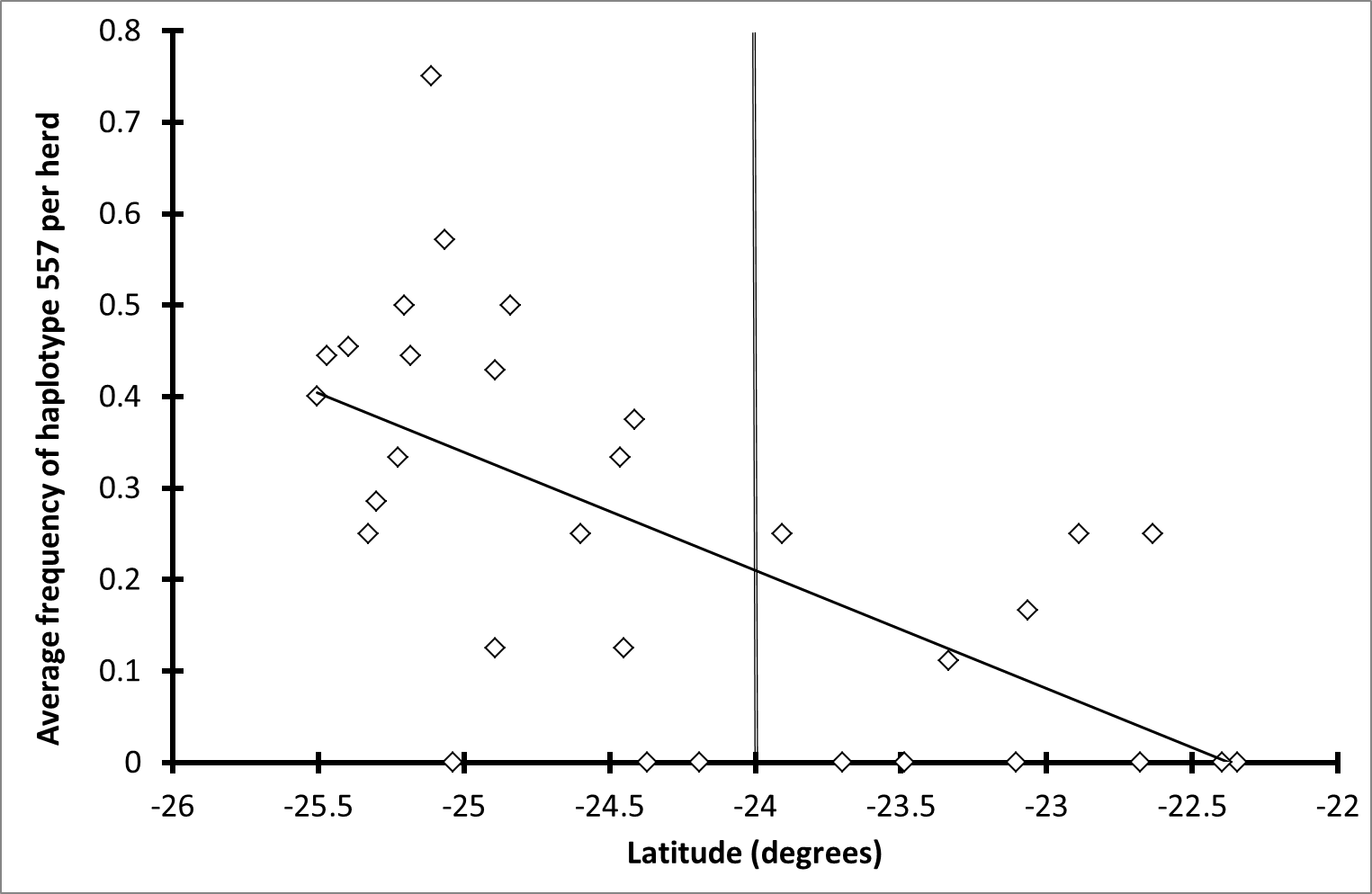


**Figure S4: Cline of Y-chromosomal haplotype 557**

Latitude < -24: southern Kruger. Y-chromosomal microsatellite data were available from one additional herd compared to the autosomal microsatellite data, the most northern one (<http://doi.org/10.5061/dryad.23d13>).

Y-chromosomal haplotype 557 showed the same spatial variation as the autosomal microsatellites: a significant latitudinal cline running in a north-south direction (*ρ* = -0.68, *P* = 0.000084, *n*_herds_ = 31, *n*_individuals_ = 201). Haplotype 557 increased in frequency from around 0.10 in the far north to around 0.40 in the far south. This cline is indicative of positive selection. Selection was apparently strong enough to maintain this cline despite a high level of male gene flow as indicated by low *F*_ST_-values in this study and by a monthly adult male dispersal rate (proportion of migrants) between herds of 0.09-0.26 [1]. A Y-chromosomal sex-ratio suppressor being the primary agent of selection, in our hypothesized mechanism of positive selection of alleles deleterious to male body condition (Figure 7), is supported by the fact that the cline of haplotype 557 was considerable stronger than the allele clines of the autosomal microsatellites, despite male dispersal being 4.5 to 13 times as high as female dispersal [1].

*References*

1. Cross PC, Getz WM (2006) Assessing vaccination as a control strategy in an ongoing epidemic: Bovine tuberculosis in African buffalo. Ecol Model 196: 494-504.
