# Supplementary material for: Positive Selection of Deleterious Alleles through Interaction with a Sex-Ratio Suppressor Gene in African Buffalo: A Plausible New Mechanism for a High Frequency Anomaly
Source: PLoS One. 2014 Nov 5;9(11):e111778. doi: 10.1371/journal.pone.0111778 (PMC4221135; doi:10.1371/journal.pone.0111778)
Supplement: Figure S5 — Regression between ML- H e per year-cohort and preconception rainfall. (DOCX) [file pone.0111778.s005.docx]

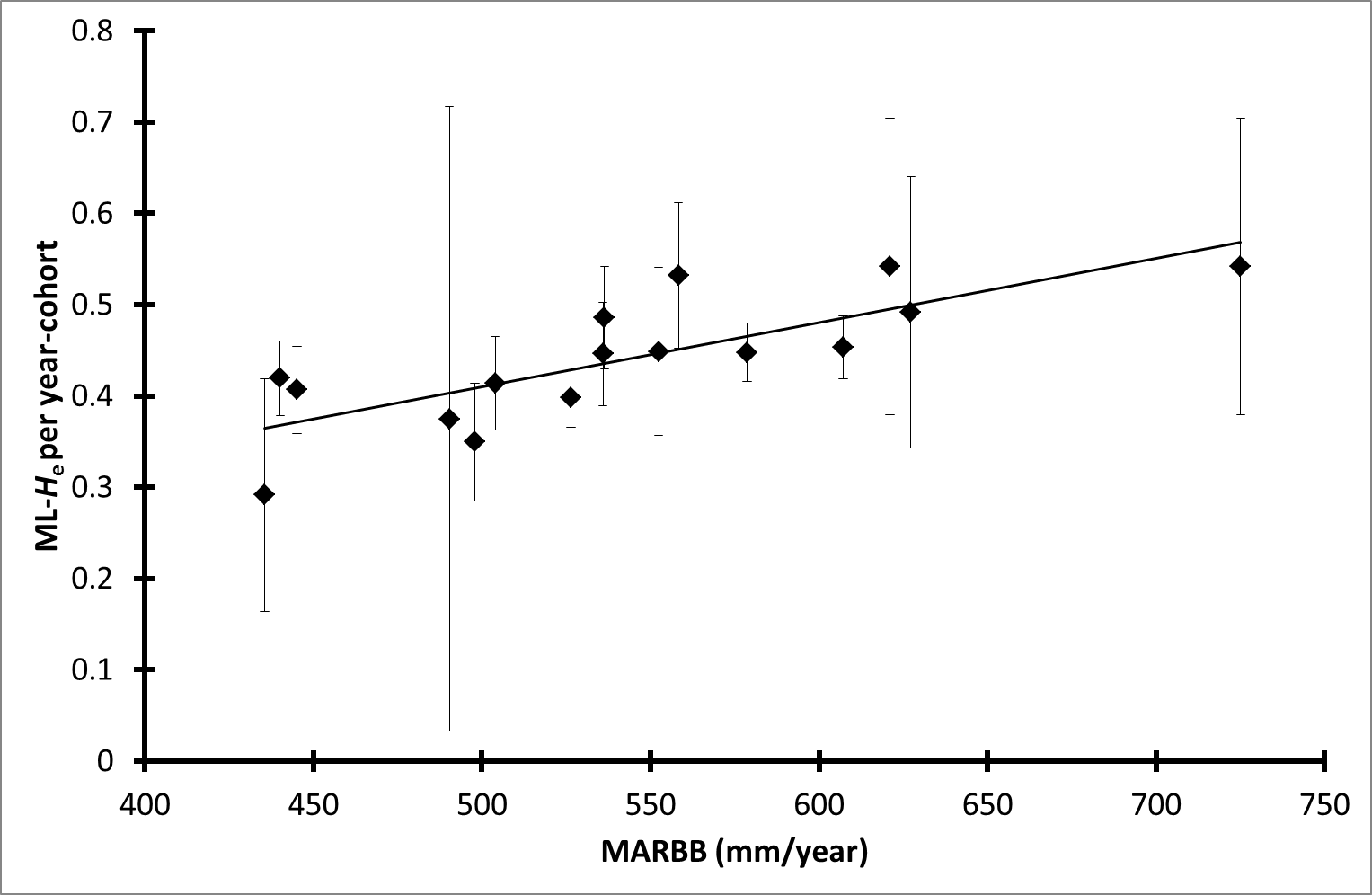


**Figure S5: Regression between ML-*H*_e_ per year-cohort and preconception rainfall**

Vertical axis: ML-*H*_e_ except for the 14-year old cohort where *n*_individuals_ = 1. Here ML-*H*_o_ was used. Error bars: 95% CI with SE derived from the average variance per microsatellite divided by the number of microsatellites, *R*_adj._^2^ = 0.59, *n*_microsatellites_ = 8 (with majority allele), *n*_cohorts_ = 16; *P*_randomization among cohorts_ = 0.0015. Data are from southern Kruger. MARBB: mean annual rainfall in the three years before birth, ML-*H*_e_: multilocus expected heterozygosity. Annual rainfall data were obtained from the South African Weather Service with years running from November to October (<http://doi.org/10.5061/dryad.23d13>).

In an earlier study, the year-cohort frequencies of Y-chromosomal haplotype 557, which were postulated to be linked to a Y-chromosomal sex-ratio suppressor, correlated in the Kruger buffalo population with rainfall that occurs for a period prior to conception, with the strongest correlations for a three-year preconception rainfall period (MARBB: mean annual rainfall in the three years before birth) [1]. This correlation is likely due to differentials in the fertility of males with a particular Y-chromosomal haplotype after extended wet and dry periods lasting multiple years [1]. The preconception rainfall association indicates that the activity of the postulated sex-ratio distorter and suppressor genes among fathers (of the year-cohorts) is related to resource availability. If these genes are somehow related to the high relative fitness of certain LBC (low body condition) individuals from southern Kruger, then one may expect an association between MARBB and ML-*H*_e_ (multilocus expected heterozygosity) per year-cohort as well, which we indeed observed.

There was a significant positive correlation between ML-*H*_e_ per year-cohort and MARBB in southern Kruger (*ρ* = 0.82, *P*_randomization among cohorts_ = 0.00071, *n*_year-cohorts_ = 16, *n*_individuals_ = 321; northern Kruger: *ρ* = -0.31, *P*_randomization among cohorts_ = 0.26, *n*_year-cohorts_ = 17, *n*_individuals_ = 138; randomization of complete individual multilocus genotypes among year-cohorts, 100,000 randomizations). The correlation was especially strong among the eight microsatellites with a majority allele (*ρ* = 0.87, *P*_randomization among cohorts_ = 0.00013; remaining nine microsatellites: *ρ* = 0.15, *P*_randomization among cohorts_ = 0.63). The latter correlation was also highly significant when each year-cohort was treated as a single data point (i.e. regular Spearman rank correlation without applying randomization, *P* = 0.0000095) or when compared against the null model, wherein allele frequencies were equalized between the year-cohorts in each herd (*P*_randomization per herd_ = 0.0014). Furthermore, the latter correlation was significantly more positive than in northern Kruger (*P*_randomization per herd among cohorts_ = 0.0029, compared against the null model). A linear regression with the microsatellites with a majority allele between ML-*H*_e_ and MARBB explained 59% (*R*^2^_adj._) of the total variation (*P*_randomization among cohorts_ = 0.0015, Figure S5). The correlation between average number of homozygous majority alleles per individual and MARBB was also significant (each year-cohort treated as a single data point, *ρ* = 0.57, *P* = 0.021).

The positive slope indicates that during dry years (preconception), when average body condition is low, parent pairs with low ML-*H*_o_ (multilocus observed heterozygosity) had a relatively high reproductive success, resulting in low ML-*H*_e_ of year-cohorts born in subsequent years. The regression between preconception rainfall, a proxy for resource availability and consequently correlated with parental body condition [1], and ML-*H*_e_ is in agreement with the observed significant logistic regression between individual body condition and ML-*H*_o_ (see main text). A strong (indirect) impact of rainfall on body condition is indicated by the observation that preconception rainfall was correlated with ML-*H*_e_ at the microsatellites with a majority allele; i.e. the microsatellites that were most strongly associated with body condition. It is further supported by a strong positive association between amount of annual rainfall and pregnancy rate in the Kruger buffalo [1]. The preconception rainfall association for both autosomal and earlier published Y-chromosomal microsatellite data [1] indicates that the activities of autosomal deleterious alleles and a Y-chromosomal sex-ratio suppressor linked to these microsatellites are not independent of each other.

*References*

1. van Hooft P, Prins HHT, Getz WM, Jolles AE, van Wieren SE, et al. (2010) Rainfall-driven sex-ratio genes in African buffalo suggested by correlations between Y-chromosomal haplotype frequencies and foetal sex ratio. BMC Evol Biol 10. Available: <http://www.biomedcentral.com/1471-2148/10/106>. Accessed 7 October 2014.
