# Supplementary material for: Positive Selection of Deleterious Alleles through Interaction with a Sex-Ratio Suppressor Gene in African Buffalo: A Plausible New Mechanism for a High Frequency Anomaly
Source: PLoS One. 2014 Nov 5;9(11):e111778. doi: 10.1371/journal.pone.0111778 (PMC4221135; doi:10.1371/journal.pone.0111778)
Supplement: Table S1 — Summary of the P -values in statistical tests for associations between body condition status and various genetic parameters. (DOCX) [file pone.0111778.s006.docx]

Table S1: Summary of the *P*-values in statistical tests for associations between body condition status and various genetic parameters

| Statistical test | Northern Kruger | Southern Kruger | Southern Kruger corrected for population stratification | North-South difference corrected for population stratification |
| --- | --- | --- | --- | --- |
| HFCs | | | | |
| Logistic regression: LBC/HBC vs. (a.o.) ML-*H*_o_ | 0.74  (n.s.) | 0.019^a^  (n.s.) | 0.048^b^  (n.s.) | 0.19  (n.s.) |
| Spearman corr.: PL-*H*_e_ LBC-HBC difference vs. baseline PL-*H*_e_ | 0.53  (n.s.) | 0.00034  (0.027) | 0.0057  (0.046) | 0.41  (n.s.) |
| Difference in mean ML-*H*_e_: LBC vs. HBC^c^ | 0.86  (n.s.) | 0.0023  (0.016) | 0.068^d^  (n.s.) | 0.31  (n.s.) |
| Majority alleles | | | | |
| χ^2^ test: homozygous majority alleles vs. other genotype classes | 0.42  (n.s.) | 0.0013  (0.0039) | 0.021  (0.042) | 0.035  (n.s.) |
| Logistic regression: LBC/HBC vs. (a.o.) homozygous majority alleles | 0.51  (n.s.) | 0.00059^a^  (0.0024) | 0.0050^b^  (0.015) | 0.30  (n.s.) |
| Sexually antagonistic alleles | | | | |
| Spearman corr.: LBC-HBC allele freq. differences males vs. females | 0.80  (n.s.) | 0.0024  (0.0047) | 0.00094  (0.0038) | 0.012  (0.049) |
| Spearman corr.: effect size vs. baseline allele frequency | 0.80  (n.s.) | 0.027  (0.027) | 0.034  (0.034) | 0.91  (n.s.) |

a: multiple logistic regression excluding herd latitude, b: multiple logistic regression including herd latitude. The weighted Holm-Bonferroni corrected *P*-values are shown between brackets. c: This test is not described in the main text of the paper. However, since it was performed by us, we decided to include it in the calculation of the Holm-Bonferroni corrected *P*-values. The differences in PL-*H*_e_ between the LBC and HBC group (the test in the row above) in southern Kruger resulted in a near-significant difference in ML-*H*_e_ between these two groups. d: The correction for population stratification probably resulted in a conservative *P*-value. The reason for this is explained at the end of the document.

In applying the weighted Holm-Bonferroni correction two families of statistical tests were used: 1) tests for HFCs and 2) tests for associations between specific alleles and body condition that underlie significant HFCs (they thus constitute a refinement of the earlier tests for HFCs). In test family 1, nine tests were applied for northern and southern Kruger and three tests for the North-South difference. For clarity, *P*-values from tests in southern Kruger that involved BTB status (northern Kruger was essentially BTB-free), which were all non-significant (*P* > 0.05), are not shown. In test family 2, four different types of tests were applied for northern and southern Kruger, and for the North-South difference.

Each test was weighted by the square root of its sample size, i.e. the number of individuals. Thus in tests involving HFCs per region relatively little weight was given to the results from northern Kruger because of small sample size (138 individuals vs. 320 individuals in southern Kruger). The weights were equal for tests involving North-South differences and for tests involving majority alleles or sexually antagonistic alleles, reducing the correction to the ordinary Holm-Bonferroni procedure.

d. A declining PL-*H*_e_ decrease in the LBC group (relative to the HBC group) with increasing baseline PL-*H*_e_ (Figure 1, second row in Table S1) was also observed in the null model (data from southern Kruger; Spearman *ρ* = 0.60, *P* = 0.011, *n*_microsatellites_ = 17; conservative estimate: *ρ* = 0.53, *P* = 0.055, *n*_microsatellites_ = 14, these are average values for all six possible combinations of excluding three of the five microsatellites in LD). As a result, PL-*H*_e_ was lower in the LBC group than in the HBC group for most microsatellites in the null model (14 out of 17, *P*_sign test_ = 0.013). This can only occur due to a negative correlation between proportion of LBC individuals per herd and ML-*H*_e_ per herd (data from southern Kruger; Spearman *ρ* = -0.44, *P* = 0.050, *n*_herds_ = 20, *n*_microsatellites_ = 17), because in the null model there were no LBC-HBC differences within herds. Observing the same pattern of LBC-HBC differences between herds as within herds is an important result, because it shows that most of the differences between herds in PL-*H*_e_, and consequently in ML-*H*_e_, are likely to be attributed to HFCs (i.e. low PL-*H*_e_ causing high LBC proportion) rather than genetic drift due to population stratification (i.e. accidental co-occurrence of low PL-*H*_e_ and high LBC proportion). Thus, correction for population stratification resulted in a conservative *P*-value because it ignored the effect of HFCs on the differences between herds.

Abbreviations: BTB: bovine tuberculosis, LBC: low body condition, LD: linkage disequilibrium, HBC: high body condition, HFC: heterozygosity-fitness correlation, ML-*H*_e_: multilocus expected heterozygosity, ML-*H*_o_: multilocus observed heterozygosity, PL-*H*_e_: expected heterozygosity per locus
