# Supplementary material for: Positive Selection of Deleterious Alleles through Interaction with a Sex-Ratio Suppressor Gene in African Buffalo: A Plausible New Mechanism for a High Frequency Anomaly
Source: PLoS One. 2014 Nov 5;9(11):e111778. doi: 10.1371/journal.pone.0111778 (PMC4221135; doi:10.1371/journal.pone.0111778)
Supplement: Table S2 — ML- H e per sex, body condition class and type of microsatellite (with or without a majority allele). (DOCX) [file pone.0111778.s007.docx]

**Table S2: ML*-H*_e_ per sex, body condition class and type of microsatellite (with or without a majority allele)**

| Sex | Body condition | ML*-H*_e_ (baseline PL-*H*_e_ < 0.56) microsatellites with majority allele | *P*_randomization_ LBC-HBC difference | ML*-H*_e_ (baseline PL-*H*_e_ > 0.75) microsatellites without majority allele | *P*_randomization_ LBC-HBC difference |
| --- | --- | --- | --- | --- | --- |
| Female | LBC | 0.426 ± 0.026 |  | 0.831 ± 0.028 |  |
|  | HBC | 0.468 ± 0.036 |  | 0.811 ± 0.036 |  |
|  | LBC minus HBC | -0.042 ± 0.044 | 0.076 | +0.020 ± 0.019 | 0.021 |
| Male | LBC | 0.398 ± 0.009 |  | 0.818 ± 0.012 |  |
|  | HBC | 0.485 ± 0.017 |  | 0.835 ± 0.015 |  |
|  | LBC minus HBC | -0.086 ± 0.046 | 0.00056 | -0.017 ± 0.019 | 0.092 |

Difference between sexes: LBC-HBC difference among females minus LBC-HBC difference among males: microsatellites with majority allele: *P*_randomization_ = 0.21, microsatellites without majority allele: *P*_randomization_ = 0.0053.

*P*-values estimated on basis of 100,000 randomizations per sex; mean ± 2SE, with SE estimated by bootstrapping (1000 X). Females: *n*_LBC_ = 138, *n*_HBC_ = 48; males: *n*_LBC_ = 92, *n*_HBC_ = 42. Data are from southern Kruger.

Abbreviations: LBC: low body condition, HBC: high body condition, ML*-H*_e_: multilocus expected heterozygosity, PL-*H*_e_: single-locus expected heterozygosity.
