# Supplementary material for: Positive Selection of Deleterious Alleles through Interaction with a Sex-Ratio Suppressor Gene in African Buffalo: A Plausible New Mechanism for a High Frequency Anomaly
Source: PLoS One. 2014 Nov 5;9(11):e111778. doi: 10.1371/journal.pone.0111778 (PMC4221135; doi:10.1371/journal.pone.0111778)
Supplement: Table S6 — Χ 2-test for differences in frequency distribution of single-locus genotypes between LBC and HBC individuals from southern Kruger (homozygotes vs. heterozygotes with a majority allele; baseline PL- H e <0.56). (DOCX) [file pone.0111778.s011.docx]

**Table S6: *Χ*^2^-test for differences in frequency distribution of single-locus genotypes between LBC and HBC individuals from southern Kruger (homozygotes vs. heterozygotes with a majority allele; baseline PL-*H*_e_ < 0.56)**

| Class of single-locus genotype | LBC observed | HBC observed | LBC expected | HBC expected | Fraction HBC  observed | Fraction HBC expected |
| --- | --- | --- | --- | --- | --- | --- |
| Homozygotes, with majority allele | 1011 | 345 | 985.20 | 370.80 | 0.25 | 0.27 |
| Heterozygotes, with majority allele | 631 | 273 | 656.80 | 247.20 | 0.30 | 0.27 |

χ^2^-value = 6.18, *P*_randomization_ = 0.012. Null hypothesis: identical frequency distribution of the different classes of single-locus genotypes among LBC and among HBC individuals. Probability was estimated by randomization (100,000X) of complete multilocus genotypes between the two body condition classes (LBC and HBC). *P*-value is the fraction of randomized data sets showing a χ^2^-value equal to or larger than the observed data. Total number of observed single-locus genotypes: number of microsatellites X number of individuals.

Abbreviations: HBC: high body condition, LBC: low body condition.
