# Supplementary material for: Positive Selection of Deleterious Alleles through Interaction with a Sex-Ratio Suppressor Gene in African Buffalo: A Plausible New Mechanism for a High Frequency Anomaly
Source: PLoS One. 2014 Nov 5;9(11):e111778. doi: 10.1371/journal.pone.0111778 (PMC4221135; doi:10.1371/journal.pone.0111778)
Supplement: Table S9 — Stouffer Z -test combining P -values of Spearman rank correlation per locus (correlation between allele frequency and latitude; baseline PL- H e <0.56). (DOCX) [file pone.0111778.s014.docx]

**Table S9: Stouffer *Z*-test combining *P*-values of Spearman rank correlation per locus (correlation between allele frequency and latitude; baseline PL-*H*_e_ < 0.56)**

| Microsatellite | Spearman correlation coefficient, *ρ* (*n*_herds_ = 30) | 1-sided *P*-value | *Z*-value |
| --- | --- | --- | --- |
| *INRA006* | -0.275 | 0.071 | 1.472 |
| *TGLA227* | -0.176 | 0.176 | 0.932 |
| *ETH225* | -0.120 | 0.264 | 0.632 |
| *BM3517* | -0.307 | 0.050 | 1.648 |
| *BM4028* | -0.325 | 0.040 | 1.752 |
| *ETH10* | -0.142 | 0.227 | 0.748 |
| *INRA128* | -0.292 | 0.058 | 1.568 |
| *TGLA263* | -0.224 | 0.117 | 1.189 |

*Z_S_* = 3.514, *P*_1-sided_ = 0.00022, *P*_2-sided_ = 0.00044, allele frequencies were estimated per herd.

All eight majority alleles were the most frequent throughout the whole of Kruger. Therefore, the allele clines cannot be attributed to a possible bias because the alleles were the most frequent in the whole of Kruger due to their relatively high frequency in the south.
