# Supplementary material for: Positive Selection of Deleterious Alleles through Interaction with a Sex-Ratio Suppressor Gene in African Buffalo: A Plausible New Mechanism for a High Frequency Anomaly
Source: PLoS One. 2014 Nov 5;9(11):e111778. doi: 10.1371/journal.pone.0111778 (PMC4221135; doi:10.1371/journal.pone.0111778)
Supplement: Table S10 — Stouffer Z -test combining P -values of Spearman rank correlation per locus (correlation between total frequency of the three most frequent alleles per locus and latitude; baseline PL- H e >0.75). (DOCX) [file pone.0111778.s015.docx]

**Table S10: Stouffer *Z*-test combining *P*-values of Spearman rank correlation per locus (correlation between total frequency of the three most frequent alleles per locus and latitude; baseline PL-*H*_e_ > 0.75)**

| Microsatellite | Spearman correlation coefficient, *ρ* (*n*_herds_ = 30) | 1-sided *P*-value | *Z*-value |
| --- | --- | --- | --- |
| *BM1824** | +0.405 | 0.987 | -2.222 |
| *CSSM19** | -0.659 | 0.00004 | 3.962 |
| *DIK20* | -0.352 | 0.028 | 1.909 |
| *TGLA159* | -0.058 | 0.380 | 0.305 |
| *TGLA57* | -0.100 | 0.300 | 0.525 |
| *BM719* | -0.468 | 0.005 | 2.607 |
| *BM3205** | -0.329 | 0.038 | 1.778 |
| *IILSTS026* | -0.290 | 0.060 | 1.556 |
| *SPS115* | -0.061 | 0.375 | 0.318 |

*Z_S_* = 3.579, *P*_1-sided_ = 0.00017, *P*_2-sided_ = 0.00034, allele frequencies were estimated per herd.

All nine pooled alleles (total frequency of the three most frequent alleles per locus) were the most frequent throughout the whole of Kruger. Therefore, the allele clines cannot be attributed to a possible bias because the pooled alleles were the most frequent in the whole of Kruger due to their relatively high frequency in the south.

*: Loci showing significant LD (between all three possible pairs)

For unlinked loci, when there is no allele cline, the probability that the Spearman correlation coefficient between latitude and allele frequency has the same sign for a pair of loci is expected to be 0.5. However, three loci were in LD, which may have increased the probability that two loci showed the same sign. However, this increase was probably only minor when considering the following two observations. First, frequencies of the pooled alleles (total of the three most frequent alleles) per herd were not significantly correlated (Spearman correlation) between the three locus pairs in LD (*BM1824-CSSM19*: *P* = 0.72, *BM1824-BM3205*: *P* = 0.42, *CSSM19-BM3205*: *P* = 0.12). Second, when randomizing coordinates among herds, between 45% and 60% of the randomizations (100,000 X) showed the same sign for the Spearman correlation coefficient between latitude and pooled allele frequency for any two loci in LD (*BM1824-CSSM19*: 49%, *BM1824-BM3205*: 45%, *CSSM19-BM3205*: 60%).
