# Supplementary material for: Positive Selection of Deleterious Alleles through Interaction with a Sex-Ratio Suppressor Gene in African Buffalo: A Plausible New Mechanism for a High Frequency Anomaly
Source: PLoS One. 2014 Nov 5;9(11):e111778. doi: 10.1371/journal.pone.0111778 (PMC4221135; doi:10.1371/journal.pone.0111778)
Supplement: Table S11 — Χ 2-test for difference in frequency distribution of homozygous majority alleles between males with and males without haplotype 557 (baseline PL- H e <0.56). (DOCX) [file pone.0111778.s016.docx]

**Table S11: *Χ*^2^-test for difference in frequency distribution of homozygous majority alleles between males with and males without haplotype 557 (baseline PL-*H*_e_ < 0.56)**

| Class of single-locus genotype | Haplotype 557  observed | Other haplotype  Observed | Haplotype 557 expected | Other haplotype expected | Fraction haplotype 557 observed | Fraction haplotype 557 expected |
| --- | --- | --- | --- | --- | --- | --- |
| Homozygotes, with majority allele | 192 | 347 | 175.63 | 363.37 | 0.36 | 0.33 |
| Other genotypes | 127 | 313 | 143.37 | 296.63 | 0.29 | 0.33 |

χ^2^-value = 5.04, *P*_randomization_ = 0.019. Null hypothesis: identical frequency distribution of the different classes of single-locus genotypes among 557-carrying males and other males. Probability was estimated by randomization (100,000X) of complete multilocus genotypes between the two classes of males (with and without haplotype 557). *P*-value is the fraction of randomized data sets showing a χ^2^-value equal to or larger than the observed data. Total number of observed single-locus genotypes: number of microsatellites X number of individuals.
