# Supplementary material for: Positive Selection of Deleterious Alleles through Interaction with a Sex-Ratio Suppressor Gene in African Buffalo: A Plausible New Mechanism for a High Frequency Anomaly
Source: PLoS One. 2014 Nov 5;9(11):e111778. doi: 10.1371/journal.pone.0111778 (PMC4221135; doi:10.1371/journal.pone.0111778)
Supplement: Text S1 — Alternative explanations. (DOCX) [file pone.0111778.s017.docx]

**Text S1: Alternative explanations**

The results of this study are unlikely to be explained by null alleles (microsatellite alleles that are present but not amplified in the PCR reaction). The results remained significant (*P* < 0.05), albeit not after correction for population stratification, when the five microsatellites with significant positive *F*_IS_ values (which may be a consequence of null alleles) were excluded from the analyses. Further, also among these microsatellites we consider it unlikely that an eventual presence of null alleles has caused biased results, since null alleles are not expected to be associated with body condition status, disease status, sex (sexual antagonism), locality (allele clines) or age (associations with preconception rainfall). Although a null allele can in principle behave as a regular allele in the sense that it can be in LD with an allele at an expressed gene, this is unlikely to occur at the majority of these microsatellites and even more unlikely to be consistently associated with deleterious effects.

As explained in the main text of the paper, the observations in this study are difficult to explain by the occurrence of genome-wide heterozygosity correlations among loci due to inbreeding; the mechanism that is assumed to underlie most occurrences of HFCs [1]. Moreover, this mechanism cannot explain the occurrence of sexual antagonism and the associations between diploid autosomal and haploid Y-chromosomal microsatellite data. We propose that the observed HFCs are due to LD between microsatellites and expressed genes. This LD is unlikely to be attributed to a small effective population size that occurred during the population bottleneck caused by the rinderpest pandemic of the late 19th century [2]. Rather, the effective population size during this bottleneck must have been quite high, considering the relatively high number of mitochondrial and Y-chromosomal haplotypes in the present-day population (34 and 15, respectively) [3,4].

The negative correlation between LBC-minus-HBC allele frequency differences in males and those in females was still significant when the age distributions were equalized between sexes by randomly deleting individuals from the largest of the two groups per year-cohort (*ρ* = -0.48, *P*_randomization per sex_ = 0.0015, *n*_LBC,females_ = 76, *n*_HBC,females_ = 45, *n*_LBC,males_ = 80, *n*_HBC,males_ = 41). The opposing allele frequency dynamics between sexes cannot be explained by alleles at expressed genes having a relatively stronger physiological effect in females (i.e. not opposite to that in males), because in that case a positive correlation between sexes would be expected. The findings also cannot be explained by sex-biased migration among herds or by immigration from northern Kruger. In the absence of selection and considering the small *F*_ST_-values in the buffalo population of Kruger, one does not expect among migrants large allele frequency differences between sexes or between the two body condition classes. However, the opposite allele frequency dynamics between sexes would require that where immigrant males have positive allele frequency deviations from residents, female immigrants have negative allele frequency deviations, and vice versa. This can only occur if male and female migrants have different geographic origins. Furthermore, consistently positive and negative allele frequency deviations cannot be explained by genetic drift. Finally, sex-biased immigration cannot explain the observed association between baseline allele frequency and effect size (LBC-HBC allele frequency differences for both sexes combined; Figure 4).

The deleterious alleles appear to be under positive rather than negative selection, as normally would be the case. In particular, negative selection cannot explain why the (autosomal) microsatellite allele clines had their lowest frequency where significant effects were absent. Not one statistical test we conducted was significant in northern Kruger. This lack of significance is not convincingly explained by the relatively small number of individuals sampled in this region, considering that the pattern of sexual antagonism in allele frequencies at the microsatellites without a majority allele and the association between preconception rainfall and ML-*H*_e_ at the microsatellites with a majority allele were significantly weaker in northern than in southern Kruger (see also Figure S5). Thus the postulated deleterious and sexually antagonistic alleles are much more likely to have been favoured by positive selection in southern Kruger than disfavoured by negative selection in northern Kruger. The allele clines cannot alternatively be explained by random genetic drift, considering their repeatability across different microsatellites. This rules out any demographic or phylogeographic explanation, without assuming some type of interaction with selection.

Positive selection of deleterious alleles indicates a high relative fitness for their carriers. Apparently, the negative effects on body condition are somehow compensated for by positive effects on fertility, because it is highly unlikely that LBC is associated with high mating success. The occurrence of allele clines, which probably need at least several generations to develop to detectable levels with our sample sizes, and a correlation between ML-*H*_e_ and preconception rainfall (Figure S5) indicates that the genetic effects on body condition were not typical just for the seasonal conditions (end dry-early wet) at the moment of sampling. Compensation of low body condition by high fertility can occur either at the individual level (i.e. deleterious alleles associated with high absolute fertility) or at the population level (i.e. deleterious alleles associated with high relative fertility). The traditional explanation would be compensation at the individual level, which does not require direct or indirect interactions with other individuals. In this explanation, the postulated deleterious alleles are under positive selection because they hitchhike with alleles that enhance fertility. However, hitchhiking with fertility-enhancing alleles fails to explain the associations between autosomal and Y-chromosomal microsatellite data, in particular the higher frequency of homozygous majority alleles among males carrying haplotype 557 than among other males. Under normal circumstances, no such associations are to be expected between autosomal and Y-chromosomal loci because they are independently inherited. For an association among a Y-chromosomal haplotype, an autosomal deleterious allele and an autosomal fertility-enhancing allele to occur (i.e. LD among three loci), the selective advantage of their co-occurrence has to be very high to overcome the fast LD decay between the Y-chromosome and each of the autosomes (a halving each generation).

Abbreviations: HBC: high body condition, HFC: heterozygosity-fitness correlation, LBC: low body condition, LD: linkage disequilibrium, ML-*H*_e_: multilocus expected heterozygosity

*References*

1. Szulkin M, Bierne N, David P (2010) Heterozygosity-fitness correlations: a time for reappraisal. Evolution 64: 1202-1217.

2. van Hooft WF, Groen AF, Prins HHT (2000) Microsatellite analysis of genetic diversity in African buffalo (*Syncerus caffer*) populations throughout Africa. Mol Ecol 9: 2017-2025.

3. van Hooft P, Greyling BJ, Prins HHT, Getz WM, Jolles AE, et al. (2007) Selection at the Y chromosome of the African buffalo driven by rainfall. PLoS ONE 2. Available: <http://www.plosone.org/article/info%3Adoi%2F10.1371%2Fjournal.pone.0001086>. Accessed 7 October 2014.

4. Greyling BJ (2007) Genetic variation, structure and dispersal among Cape buffalo populations from the Hluhluwe-Imfolozi and Kruger National Parks of South Africa [PhD thesis]. Pretoria: University of Pretoria. 205 p.
